# Synaptic Homeostasis and Restructuring across the Sleep-Wake Cycle

**DOI:** 10.1371/journal.pcbi.1004241

**Published:** 2015-05-28

**Authors:** Wilfredo Blanco, Catia M. Pereira, Vinicius R. Cota, Annie C. Souza, César Rennó-Costa, Sharlene Santos, Gabriella Dias, Ana M. G. Guerreiro, Adriano B. L. Tort, Adrião D. Neto, Sidarta Ribeiro

**Affiliations:** 1 Brain Institute, Federal University of Rio Grande do Norte, Natal, Rio Grande do Norte, Brazil; 2 Department of Computer and Automation, Federal University of Rio Grande do Norte, Natal, Rio Grande do Norte, Brazil; 3 Department of Computer Science, State University of Rio Grande do Norte, Natal, Rio Grande do Norte, Brazil; 4 Edmond and Lily Safra International Institute of Neuroscience of Natal (ELS-IINN), Natal, Rio Grande do Norte, Brazil; 5 Laboratory of Neuroengineerging and Neuroscience, Federal University of São João Del-Rei, São João Del-Rei, Minas Gerais, Brazil; 6 Department of Biomedical Engineering, Federal University of Rio Grande do Norte, Natal, Rio Grande do Norte, Brazil; Imperial College London, UNITED KINGDOM

## Abstract

Sleep is critical for hippocampus-dependent memory consolidation. However, the underlying mechanisms of synaptic plasticity are poorly understood. The central controversy is on whether long-term potentiation (LTP) takes a role during sleep and which would be its specific effect on memory. To address this question, we used immunohistochemistry to measure phosphorylation of Ca^2+^/calmodulin-dependent protein kinase II (pCaMKIIα) in the rat hippocampus immediately after specific sleep-wake states were interrupted. Control animals not exposed to novel objects during waking (WK) showed stable pCaMKIIα levels across the sleep-wake cycle, but animals exposed to novel objects showed a decrease during subsequent slow-wave sleep (SWS) followed by a rebound during rapid-eye-movement sleep (REM). The levels of pCaMKIIα during REM were proportional to cortical spindles near SWS/REM transitions. Based on these results, we modeled sleep-dependent LTP on a network of fully connected excitatory neurons fed with spikes recorded from the rat hippocampus across WK, SWS and REM. Sleep without LTP orderly rescaled synaptic weights to a narrow range of intermediate values. In contrast, LTP triggered near the SWS/REM transition led to marked swaps in synaptic weight ranking. To better understand the interaction between rescaling and restructuring during sleep, we implemented synaptic homeostasis and embossing in a detailed hippocampal-cortical model with both excitatory and inhibitory neurons. Synaptic homeostasis was implemented by weakening potentiation and strengthening depression, while synaptic embossing was simulated by evoking LTP on selected synapses. We observed that synaptic homeostasis facilitates controlled synaptic restructuring. The results imply a mechanism for a cognitive synergy between SWS and REM, and suggest that LTP at the SWS/REM transition critically influences the effect of sleep: Its lack determines synaptic homeostasis, its presence causes synaptic restructuring.

## Author Summary

Sleep is important for long lasting memories. There exists, however, a controversy regarding the mechanisms by which sleep modifies synapses to consolidate enduring memories. One theory posits that sleep weakens synapses, leading to the forgetting of all but the strongest memories. The alternative theory proposes that sleep promotes both weakening and strengthening of different connections, the latter through a process known as long-term potentiation (LTP). Here we measured the levels of a phosphorylated protein related to LTP during the sleep cycle of rats and used the data to build models of sleep-dependent synaptic plasticity. By feeding one model with spikes recorded from the rat hippocampus, we observed that LTP during sleep not merely strengthens certain connections, but actually reorganizes how these connections are ranked in strength, leading to substantial changes of the overall pattern. A more detailed model of hippocampus and cortex showed that the interaction of the mechanisms predicted by the competing theories promotes a more efficient control of which memories are stored. Our results provide a step forward in the understanding of the cognitive role of sleep by indicating that the current competing theories are not mutually exclusive. Instead, each constitutes an important stage of memory consolidation.

## Introduction

In the hippocampus, slow-wave sleep (SWS) is characterized by large amplitude, low-frequency oscillations of the local field potential (LFP), concomitant with a phasic regime of neuronal firing, with relatively low mean firing rates and intermittent synchronization [1–4]. In contrast, rapid-eye-movement sleep (REM) displays small amplitude, high-frequency oscillations that underlie a tonic firing regime, with relatively high mean firing rates and decreased synchrony [1–4]. Both sleep states play a role in the consolidation of hippocampus-dependent memories [5, 6], but the mechanisms remain poorly understood.

Two theories are in dispute. The synaptic homeostasis hypothesis (SHY) proposes that SWS causes generalized synaptic weakening [7–9], leading to the down-selection of weak synapses [10]. The notion that synaptic depression is determinant for off-line memory processing departs from the conventional Hebbian learning rule, by which connections among simultaneously firing neurons are reinforced [11]. On the other hand, the synaptic embossing hypothesis postulates the combination of non-Hebbian rescaling and Hebbian potentiation of synaptic weights in complementary circuits during REM [6, 12, 13]. The core of the dispute is the controversy on whether long-term potentiation (LTP) occurs during sleep. Empirical studies diverge considerably, with molecular, electrophysiological and morphological evidence for [14–22] and against [4, 7, 23–25] it.

The theories also differ on the roles of the different sleep states in memory consolidation. SHY only considers SWS and does not propose any role for REM [4, 26], while the embossing theory encompasses both states [6, 12, 13]. The substantial differences in the firing and correlation regimes of SWS and REM suggest that the two states should be separately and sequentially modeled [6, 12, 27]. One study has suggested that SWS leads to general memory reinforcement while REM leads to forgetting of all but the strongest memories traces [28]. LTP during SWS has been proposed to amplify the synaptic changes acquired during WK [29], with further processing of the resulting synaptic weights during REM [30, 31]. In fact, plasticity factors such as protein kinases and transcription factors encoded by immediate-early genes are up-regulated during REM [14–16, 20, 32, 33]. Therefore, it is possible that a complete sleep cycle traversing SWS and REM leads to important perturbations in the pattern of synaptic weights [6, 12, 27], rather than to the simple weight convergence observed during SWS alone [4].

To address this debate, we first assessed phosphorylated Ca^2+^/calmodulin-dependent protein kinase II (pCaMKIIα) in the hippocampus of rats exposed (or not) to novel objects, and killed immediately after subsequent WK, SWS or REM (**S1 Fig**). CaMKIIα phosphorylation is one of the earliest mechanisms with a critical role in LTP, memory and learning [34, 35]. CaMKIIα undergoes conformational changes towards the active form within seconds after the beginning of synaptic stimulation [36], triggering later events that include the up-regulation of immediate-early genes required for long-term synaptic remodeling, such as Zif-268 [37]. Given the very fast kinetics of CaMKIIα phosphorylation [36] in comparison with Zif-268 [38], pCaMKIIα levels were hypothesized to show experience-dependent changes immediately after SWS and/or REM, while Zif-268 protein levels were expected to be invariant immediately after any given state. The protein levels of total CaMKIIα and Actin were also assessed as negative controls expected to show invariant levels across groups, given their much slower transcriptional and translational regulation. To gain insight in the state dependency of pCaMKIIα regulation, we also investigated the relationship between pCaMKIIα levels and electrophysiological markers of SWS (delta oscillations), REM (theta oscillations) or the SWS/REM transition (neocortical spindles) [39].

Since both SHY and the synaptic embossing theory have empirical support, computational work may be particularly insightful. SHY has been modeled at the single neuron and network levels, but without real neurophysiological inputs [4, 10]. A recent SHY model found deleterious effects for memory when instantaneous potentiation was switched on during sleep [10], but the synaptic embossing hypothesis is yet to be simulated with realistic LTP onset and dynamics. To that end, we computationally investigated the network consequences of LTP triggered during sleep, by feeding simulated neurons with action potentials recorded from the rat hippocampus across the sleep-wake cycle. LTP was applied at REM onset as constrained by the empirical results. Synchronization-based LTP was calculated from coincident spiking during SWS only or SWS+REM, while an alternative model captured the notion that short-term changes in pCaMKIIα levels determine long-term increases in synaptic weights. Finally, we investigated the interaction of synaptic homeostasis and embossing mechanisms by simulating the dynamics of memory formation during a sleep cycle in a canonical hippocampo-cortical model.

## Materials and Methods

### Ethics Statement

For quantification of pCaMKIIα and Zif-268 levels, adult male Wistar rats (n = 27, 300–350 g) were housed, surgically implanted and recorded according to National Institutes of Health (NIH) guidelines and Edmond and Lily Safra International Institute of Neuroscience of Natal (ELS-IINN) Committee for Ethics in Animal Experimentation (permit # 05/2007). Implanted animals were housed in individual home cages with food and water *ad libitum*, and were kept on a 12h light/dark schedule with lights on at 06:00. At the end of the experiment, rats were anesthetized with isoflurane 5%, and decapitated.

### Experimental Protocol

#### Implants and electrophysiological recordings

For the LFP recordings, rats were implanted with monopolar LFP electrodes for cortical and reference leads, and microwires in the dentate gyrus. LFPs were recorded with a 32-channel multichannel acquisition processor, synchronized with video recordings and animal tracking (Plexon, USA). See S1 Text. Real spike data, used as inputs to the fully connected excitatory network, were previously obtained by chronic extracellular multi-electrode recordings from hippocampal CA1 field (n = 6 rats; same data as in [20, 40]).

#### Novel object exploration

Animals were transferred to the recording box at 07:00 pm. After cycling freely across sleep-wake states for 2h, 4 different novel objects were placed in the box corners for 10 min [31] (S1A Fig). Controls were not exposed to novel objects (S1B Fig).

#### State sorting with spectral maps

Precise determination of sleep-wake states was obtained by real time spectral analysis of LFP. Customized Matlab routine was used to build a 2D spectral map that sorts WK, SWS and REM in real time [20, 39]. On experiment day, 6 groups were defined by prior experience (with or without exploration of novel objects) and state displayed immediately before killing (WK, SWS or REM). See S1 Fig and S2 Text. Following object exploration, rats were kept awake by gently tapping the recording box every time they approached SWS on the online spectral map. This mild sleep deprivation was used to temporally disambiguate the changes induced by novel object exploration from the subsequent sleep-dependent changes in pCaMKIIα levels. By keeping all the animals awake for 3h, overall behavioral experience was equalized across groups and therefore the specific sleep-wake state achieved immediately before killing became the key variable to consider. We have successfully employed this procedure in the past for the assessment of other plasticity factors during post-learning sleep [14, 20, 32, 41].

#### Group assignment

After 3h the animals were randomly assigned to the WK, SWS or REM groups (S1 Fig). SWS animals were killed immediately after 10 min of uninterrupted SWS; REM animals were killed immediately after 2 min of uninterrupted REM; WK animals were kept awake for additional 10 min before killing. Group assignment was identically performed for unexposed controls.

#### Quantification of protein levels

Labeling specificity of the immunohistochemistry for pCaMKIIα, total CaMKIIα, Actin and Zif-268 (S2A Fig) was confirmed by Western blots (S2B Fig). Quantification of the immunohistochemistry comprised 2 sections per animal, with all sections processed as a single batch (n = 5 for groups exposed to objects, n = 4 for control groups). See S3 and S4 Text.

#### Spindle quantification

Customized Matlab routines were used to quantify spectral power in the delta (0.5 to 4.5 Hz) and theta (4.5 to 12 Hz) bands, and to detect and quantify spindles, 7–14 Hz oscillations that last from 0.5 to 4 seconds [42]. As expected [39], spindles were detected during SWS and during the intermediate sleep (IS) state that separates SWS from REM [43]. See S5 Text.

### Fully Connected Excitatory Network Model (Model 1)

The network was implemented as a modified Boltzmann machine [44]. The total synaptic current for each neuron *i* is defined as $I_{i}(t)=w_{ie}e_{i}(t)+\frac{1}{N-1}\sum_{j=1}^{N}w_{ij}v_{j}(t)$ where *N* is the number of neurons, *e*_*i*_ *(t)* is an external input and *v*_*j*_(*t*) is the state of pre-synaptic neuron *j*; *w*_*ie*_ and *w*_*ij*_ are the corresponding synaptic weights. The neuron state is binary (0 or 1) and stochastically updated by the adjusted sigmoid function $P(v_{i}(t)=1)=\frac{1}{1+e^{\left(K_{t}-K_{s}I_{i}\right)}}$, where *K*_*t*_ *= 6* and *K*_*s*_ *= 11* are constants; with these values the mean firing rate of the spontaneous network activity (when *w*_*ie*_ = *0*) is around 0.5 and 1Hz (**S9 Fig**). During all simulations, we used *w*_*ie*_ = *0*.*5* and pre-synaptic weights *w*_*ij*_ were randomly initiated following a uniform distribution between 0 and 1, except when *i* = *j*, in which case the synaptic weight is set to 0 throughout the simulation.

The network was exposed to 2 types external inputs (*e*_*i*_ *(t)*): Real spike data and Poisson spike trains with same mean firing rates as those observed during WK, SWS and REM (**S4A Fig**). When Poisson spike trains were used as inputs, the network was composed of 150 neurons. When real spike data were used, the network was composed by the same number of neurons as recorded in each animal (Rat1 = 45, Rat2 = 39, Rat3 = 39, Rat4 = 34, Rat5 = 22 and Rat6 = 13). Despite the lack of inhibitory neurons, this simplified network replicates the synaptic rescaling dynamics (see below) of a more complex network with both excitatory and inhibitory processing units [4].

#### Instantaneous synaptic plasticity

Instantaneous plasticity was adapted from the stable Hebb's rule for synchronous firing [45]. Discrete synaptic potentiation (when the pre- and post- synaptic neurons fire synchronously) is described as:

$$w_{ij}(t+\Delta t)=w_{ij}(t)+{\text{C}}_{\text{p}}\;J(w_{ij}(t))\;{\text{v}}_{\text{i}}(t)v_{j}(t)\Delta \;\text{t}$$ (1)

$$J(x)=e^{-x}-e^{-1}$$ (2)

where *∆t* = 0.004s and *C*_*p*_ *=* 6.25s^-1^; notice that *J* makes the potentiation larger for weak than strong synapses [46–48]. The synaptic weakening (when the pre-synaptic neuron fires and the post-synaptic neuron does not fire) is described as:

$$w_{ij}(t+\Delta t)=w_{ij}(t)+{\text{C}}_{\text{d}}\;(v_{i}(t)-\theta )v_{j}(t)\Delta t$$ (3)

where θ = 1 and *C*_*d*_ = 0.021s^-1^.

#### LTP models during sleep

In order to model LTP during sleep, we added a Gaussian curve to Eq 1 [48, 49], which is a function of time with the following parameters: t_T_, the moment the Gaussian is triggered (30s after the beginning of the selected REM sleep) (S8 Fig); μ, the peak time (30 min); and σ the variance. The synaptic potentiation is thus defined as:

$$w_{ij}(t+\Delta t)=w_{ij}(t)+{\text{C}}_{p}\text{J}\left(w_{ij}(t)\right)v_{i}(t)v_{j}(t)\Delta t+\frac{Cieg_{ij}}{\sigma \sqrt{2\pi }}e^{\left(-\frac{{\left(t-\left(t_{T}+\mu \right)\right)}^{2}}{2\sigma^{2}}\right)}$$ (4)

where *Cieg*_*ij*_ models the effect of immediate-early genes by modulating the amplitude of the Gaussian gain. *Cieg*_*ij*_ was calculated in 2 ways: based on spike synchronization (LTP1) and on the trajectory of synaptic weights (LTP2). In LTP1, *Cieg*_*ij*_ is the ratio of presynaptic spikes that occurred synchronously with postsynaptic spikes during specific sleep stages (**S10A Fig**). Three variations of LTP1 were implemented depending on which epoch *Cieg*_*ij*_ was computed for: using the entire SWS prior to the REM stage (LTP1 full SWS); the last 30s of SWS prior to REM (LTP1 30s SWS end); and the last 30s of SWS and the first 30s of REM (LTP1 60s SWS/REM). In LTP2, synaptic connections were selected based on the angle β_ij_ formed by *w*_*ij*_ *(t)* at the SWS/REM transition; β_ij_ was obtained from the linear fit of *w*_*ij*_ *(t)* values 30s before and after the transition (**S10B and S10C Fig**). Two variations of LTP2 were implemented: selecting for potentiation in all *w*_*ij*_ *(t)* trajectories with a positive REM slope, with β_ij_ smaller than 3π/2 (LTP2 permissive 60s SWS/REM) (**S10B Fig**, left panel, dark blue dots, cases 1, 2 and 3); and selecting only trajectories with β_ij_ smaller than π (LTP2 restrictive 60s SWS/REM) (**S10B Fig**, left panel, light blue dots, cases 1 and 2).

#### Net synaptic weight changes

Net synaptic weight changes (*M*_*∆w*_) was calculated as the mean of the differences (*element by element*) between the synaptic weight values at time *t*_*1*_ and *t*_*2*_ *(t*_*2*_ *>t*_*1*_ *)*: MΔw=∑iN∑jN(wij(t2)−wij(t1))N(N−1). Since *w*_*ii*_ *= 0*, *N(N-1)* is the number of connections among neurons. Global synaptic downscale is defined as *M*_*∆w*_ *<0*; global synaptic upscale occurs when *M*_*∆w*_ *>0*.

#### Steady-state

The network was considered to reach the convergence point when the absolute variation over time (slope) of every synaptic connection value reached a negligible value, i.e. when *|dw*_*ij*_ */dt|< = ε*, *ε = 0*.*00025 for ∀i and ∀j*.

### Canonical Hippocampal-Cortical Model (Model 2)

We built a network of 45 leaky integrate-and-fire neurons with network feedback inhibition [50–52] (**S6 Text**). Each neuron received synaptic connections from 200 input units and was not directly connected to other neuron. Half of the input units were assigned to memory A (M_A_ = [1 … 100]) and the other half to memory B (M_B_ = [101 … 200]). Input unit *i* emitted actions potentials (S[t] = 1 if there is a spike at time t, S[t] = 0, otherwise) following a Poisson distribution with mean frequency f_active_ = 20 Hz when in an active state and f_inactive_ = 10 Hz when in an inactive state. Input pattern was determined in 125 ms windows by randomly selecting one memory to be in an active state. Each cell gets selective to a specific memory through the synaptic weight dynamics driven by the plasticity mechanisms described below.

#### Synaptic plasticity in the canonical network

Input-neuron synapse conductance, W_ij_[t], between input unit **j** and neuron **i** was subject to modification according to spike-timing dependent plasticity (STDP) and LTP effects. STDP was based on the relation, $\delta t_{ij}[t]=t_{pos}^{i}-t_{pre}^{j}$, between the time of the last presynaptic spike, $t_{pre}^{j}$ where $S_{pre}^{j}[t_{pre}^{j}]=1$, and the time of the last postsynaptic spike, $t_{pos}^{i}$ where $S_{pos}^{i}[t_{pos}^{i}]=1$. Potentiation, $W_{P}^{ij}[t]$, occurred when *δt*_*ij*_ > 0 and depression, $W_{D}^{ij}[t]$, when *δt*_*ij*_ < 0, and are described by:

WPij[t]=Sposi[t](Cp+vWij[t])e−δtijτSTDP (5)

WDij[t]=Sprej[t]((−Cd+v)Wij[t])eδtijτSTDP (6)

Where *τ*_*STDP*_ = 20 ms, *C*_*p*_ = 3.2 nS, *C*_*d*_ = 0.03 and *v* is a stochastic factor with zero mean and *σ* = 0.015.

LTP, $W_{LTP}^{ij}[t]$, was implemented as a Gaussian function with peak at $t_{LTP}^{peak}$, standard deviation of $t_{LTP}^{std}$ = 45 s. $W_{LTP}^{ij}[t]$ is modulated by a gain factor specific for each synapse, $\theta_{LTP}^{ij}[t]$, and a magnitude parameter *κ*_*sleep*_:

WLTPij[t]=κsleepθLTPij[t]Ae−(t−tLTPpeak)22(τLTPstd)2 (7)

Where A is such that the integral of Ae−(t−tLTPpeak)22(τLTPstd)2 equals 0.05 nS.

The sum of all plastic events, $\Delta W_{ij}[t]=W_{P}^{ij}[t]+W_{D}^{ij}[t]+W_{LTP}^{ij}[t]$, updated the weight values, *W*_*ij*_[*t* + 1]. To ensure weight stability, the sum of all weights was kept stable with a mean value of *ω* = 0.25 nS:

$$W_{ij}[t+1]=\frac{W_{ij}[t]+\Delta W_{ij}[t]}{m^{-1}\sum_{k=1}^{m}\left(W_{ik}[t]+\Delta W_{ik}[t]\right)}\cdot \omega$$ (8)

#### Simulation of sleep

Sleep was implemented through three mechanisms: 1) reduction of input frequency; 2) modulation of STDP and 3) modulation of LTP. Sleep simulation started at *t*_*sleep*_ = 315 s and ended at *t*_*wake*_ = 585 s.

During sleep the frequency of presynaptic spikes was reduced:

$$\begin{array}{cc} f_{active}^{sleep}=0.5f_{active}; & f_{inactive}^{sleep}=0.5f_{inactive}; \end{array}$$ (9)

STDP was modified during sleep through the modulation of the constant factors *C*_*P*_ and *C*_*d*_ by a factor *γ*_*sleep*_:

$$\begin{array}{cc} C_{P}^{sleep}=\frac{C_{p}}{\gamma_{sleep}}; & C_{d}^{sleep}=C_{d}\gamma_{sleep}; \end{array}$$ (10)

LTP was triggered during sleep by setting $t_{LTP}^{peak}$ = 405 s. LTP was used to influence memory selectivity of each neuron. The intended selectivity pattern in the network was a permutation of the original pattern prior to sleep. Specifically, the gain factor, $\theta_{LTP}^{ij}[t]$, depends on the normalized synaptic weight between input *j* and a randomly picked neuron *k* ≠ *i* before *t*_*sleep*_:

$$\theta_{LTP}^{ij}[t]=\frac{W_{kj}[t_{sleep}-1]}{m^{-1}\sum_{x=1}^{m}W_{kx}[t_{sleep}-1]}H\left(t-t_{sleep}\right)$$ (11)

where *H*(⋅) is a Heaviside function.

#### Memory analysis

The memory selectivity of a given neuron i at time t, *Sel*_*i*_[*t*], was set as 1 for memory A, -1 for memory B and 0 if there is no memory selectivity:

$$Sel_{i}[t]=\{\begin{array}{ccc} 1 & if & \sum_{j\in M_{A}}W_{ij}[t]>\sum_{j\in M_{B}}W_{ij}[t]\\ -1 & if & \sum_{j\in M_{A}}W_{ij}[t]<\sum_{j\in M_{B}}W_{ij}[t]\\ 0 & if & \sum_{j\in M_{A}}W_{ij}[t]=\sum_{j\in M_{B}}W_{ij}[t] \end{array}$$ (12)

The memory selectivity of neuron *i* was considered to switch during sleep when *Sel*_*i*_[*t*_*sleep*_]⋅*Sel*_*i*_[∞] = −1 and was considered stable when *Sel*_*i*_[*t*_*sleep*_]⋅*Sel*_*i*_[∞] = 1. The proportion of switches in the memory selectivity,*T*_*S*_, was measured as:

$$T_{S}=\frac{\sum_{i=1}^{45}\left(-Sel_{i}[t_{sleep}]Sel_{i}[\infty ]\right)H\left(-Sel_{i}[t_{sleep}]Sel_{i}[\infty ]\right)}{45}$$ (13)

The memory selectivity imposed by LTP to neuron *i*, $Sel_{i}^{\theta }[t]$, was set such that:

$$Sel_{i}^{\theta }[t]=\{\begin{array}{ccc} 1 & if & \sum_{j\in M_{A}}\theta_{LTP}^{ij}[t]>\sum_{j\in M_{B}}\theta_{LTP}^{ij}[t]\\ -1 & if & \sum_{j\in M_{A}}\theta_{LTP}^{ij}[t]<\sum_{j\in M_{B}}\theta_{LTP}^{ij}[t]\\ 0 & if & \sum_{j\in M_{A}}\theta_{LTP}^{ij}[t]=\sum_{j\in M_{B}}\theta_{LTP}^{ij}[t] \end{array}$$ (14)

For each neuron *i*, we call an “LTP hit” when $Sel_{{}_{i}}^{\theta }[t_{sleep}]\cdot Sel_{i}[\infty ]=1$. The proportion of LTP hits, T_H_, was measured as:

$$T_{H}=\frac{\sum_{t=1}^{45}\left(Sel_{i}^{\theta }[t_{sleep}]Sel_{i}[\infty ]\right)H\left(Sel_{i}^{\theta }[t_{sleep}]Sel_{i}[\infty ]\right)}{45}$$ (15)

The significance of T_S_ and T_H_ was assessed using a normal fit to the distribution of 200 surrogate values obtained from shuffling of the memory selectivity pattern in the network after sleep.

## Results

### Experience-Dependent CaMKIIα Phosphorylation During REM Is Proportional to Spindle Counts at the SWS/REM Transition

**Fig 1:**
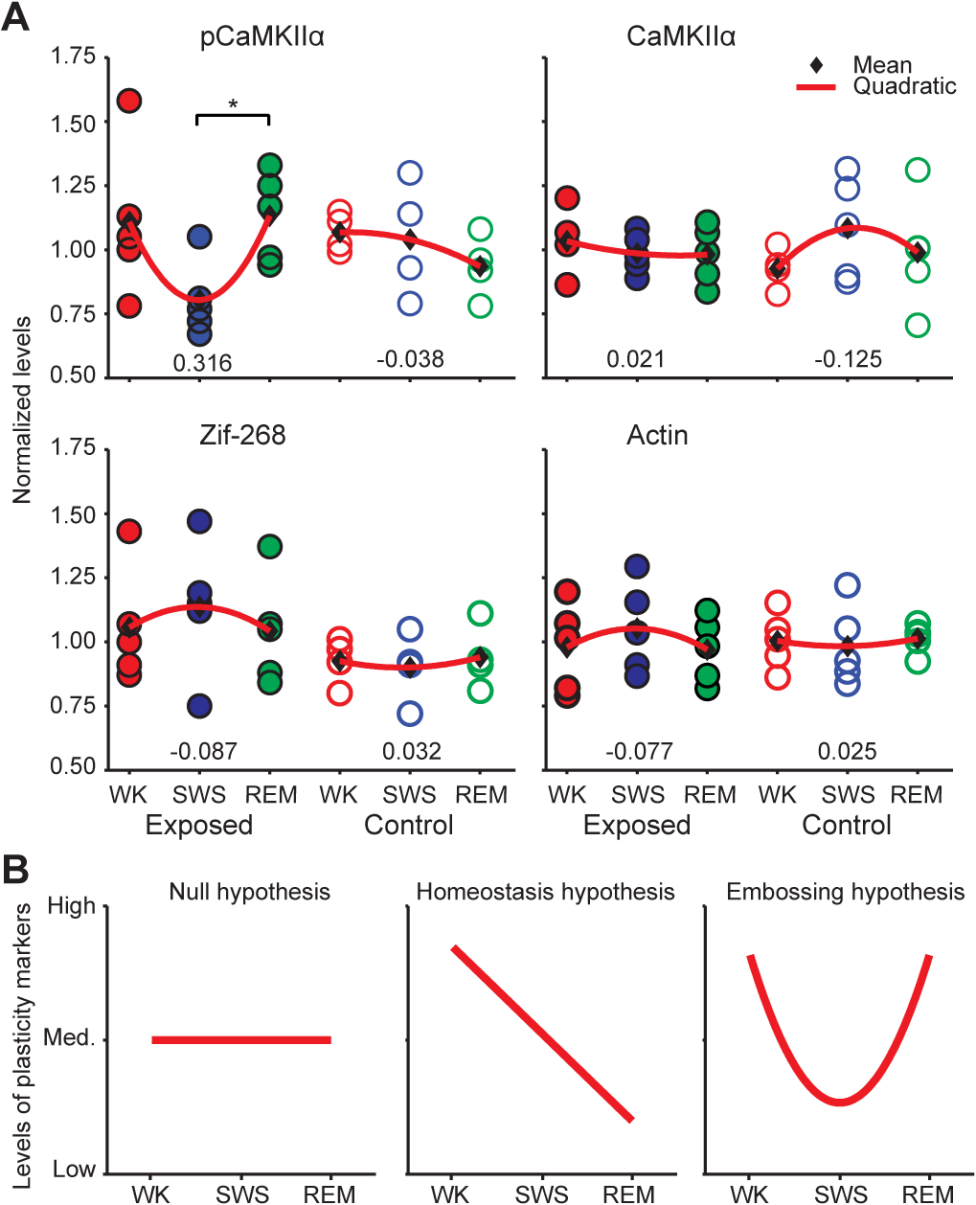
Experience-dependent CaMKIIα phosphorylation in the hippocampus during REM. (A) Normalized hippocampal levels of pCaMKIIα, total CaMKIIα, Zif-268 and Actin (mean densitometric levels, one datapoint per animal). Kruskal-Wallis followed by Dunn’s test between consecutive states revealed a significant increase from SWS to REM (*p<0.05). Normalized levels of total CaMKIIα, Zif-268 and Actin in the hippocampus were not significantly different across states. This was expected because of the slower regulation kinetics of these proteins, in comparison with pCaMKIIα. Numbers on the bottom indicate the curvatures of the quadratic fits in red. (B) Schematic representation of the theoretical predictions for levels of plasticity markers across wake-sleep states. Compare with the quadratic fits on panel A.

Six groups were defined based on prior experience (with or without exploration of novel objects), and on the state immediately before killing (WK, SWS or REM) (**S1 Fig**). In unexposed control groups, no significant differences in hippocampal pCaMKIIα levels were detected across states (**Fig 1A**; Kruskal-Wallis p = 0.2958, Dunn’s test for consecutive states, adjusted p values: WK vs. SWS p>0.9999; SWS vs. REM p = 0.5615). However, in animals previously exposed to novel objects, hippocampal CaMKIIα phosphorylation significantly increased from SWS to REM (**Fig 1A**; Kruskal-Wallis p = 0.0396, Dunn’s test, adjusted p values: WK vs. SWS p = 0.0954; SWS vs. REM p = 0.0473). The number of cells with pCaMKIIα somatic labeling was counted to determine whether the densitometric changes observed could be attributed to a sheer increase on the total number of cells engaged in the pCaMKIIα response during REM. No significant differences were detected when labeled cells were counted. This indicates that the changes observed from SWS to REM in exposed animals did not reflect increased numbers of responsive neurons, but rather increased pCaMKIIα levels in the neuropil and soma.

To control for potential *a priori* inter-group differences in baseline CaMKIIα phosphorylation levels, we assessed the protein levels of total CaMKIIα, Actin and Zif-268 in adjacent brain sections. The rationale for this comparison was the fact that the regulation of these proteins is downstream of CaMKIIα phosphorylation, with much slower kinetics [48, 49, 53]. Since killing occurred immediately after specific sleep-wake states, we did not expect the levels of total CaMKIIα, Zif-268 or Actin to be differentially modulated across groups, unless there were spurious inter-group differences unrelated to states. We found no significant differences across groups for these proteins, irrespective of previous exposure to novel objects (**Fig 1A;** Kruskal-Wallis: Zif-268 Exposed p = 0.5387, Zif-268 Control p = 0.8775; total CaMKIIα Exposed p = 0.8270, total CaMKIIα Control p = 0.6505; Actin Exposed p = 0.6907; Actin Control p = 0.8781). The even distribution of the levels of total CaMKIIα, Zif-268 or Actin across groups indicated that the increase in CaMKIIα phosphorylation from SWS to REM was not an artifact of group sorting.

It is clear that the significant increase of CaMKIIα phosphorylation from SWS to REM could only occur because of a reduction from WK to SWS, which nevertheless only reached a near-significant trend (p = 0.0954). To grasp this effect, we calculated quadratic fits of the data across wake-sleep states (**Fig 1A**, red curves, curvatures indicated by numbers in the bottom). The pCaMKIIα data show a “U” effect in the exposed group but not in the control group, while no such effect was observed for total CaMKIIα, Actin or Zif-268. Schematic pCaMKIIα profiles predicted by the synaptic homeostatic hypothesis (monotonic decrease), the synaptic embossing hypothesis (“U”shape) and the null hypothesis (stable levels) are shown in **Fig 1B**. The data fit the picture of homeostatic rescaling during SWS (decrease in pCaMKIIα levels) followed by experience-dependent LTP during REM (increase in pCaMKII levels).

To investigate the electrophysiological correlates of CaMKIIα phosphorylation during REM, we assessed the correlation of pCaMKIIα levels with LFP power in the delta (0.5 to 4.5 Hz) and theta (4.5 to 12 Hz) bands during the last 15 min before killing. Neither frequency band showed significant correlations with pCaMKIIα levels (for theta, SWS group: R^2^ = 0.05322 and p = 0.5504, REM group: R^2^ = 0.02431 and p = 0.6888; for delta, SWS group: R^2^ = 0.002432 and p = 0.8997, REM group: R^2^ = 0.1463 and p = 0.3097).

**Fig 2:**
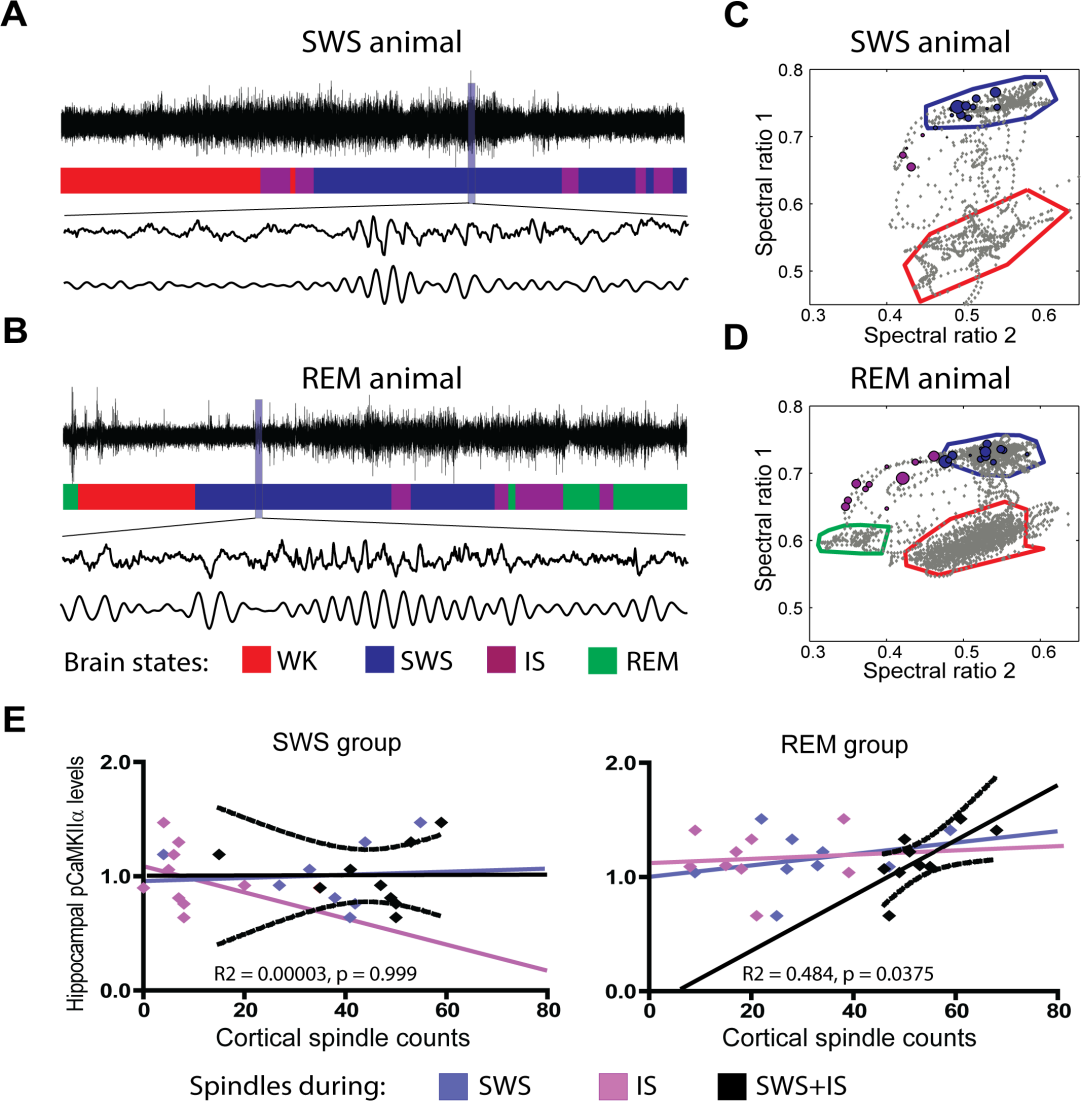
Cortical spindles at the SWS/REM transition correlate with CaMKII phosphorylation in the hippocampus. (A) Raw LFP recordings of representative SWS animal (top trace) classified according to state (colored hypnogram). Cortical spindles before (middle trace) and after band-pass filtering in the spindle range (bottom trace). (B) Same as (A) for representative REM animal. (C) Spectral map of representative SWS animal, depicting sleep-wake cycle with state clusters and borders. Cortical spindles marked as circles of size proportional to duration, color-coded by hypnogram. (D) Same as (C) for representative REM animal. (E) Cortical spindle counts and hippocampal pCaMKIIα were significantly correlated in the REM group when all spindles were considered (R^2^ = 0.484, p = 0.0375), i.e. pooled from SWS and IS. No correlation was observed during SWS only (R^2^ = 0.116, p = 0.369) or IS only (R^2^ = 0.007, p = 0.825). No correlation was observed in the SWS group for SWS+IS (R^2^ = 0.00003, p = 0.999), SWS only (R^2^ = 0.006, p = 0.849) or IS only (R^2^ = 0.052, p = 0.5560). Plots include both exposed and control animals. See also **S1, S2 Figs**.

Next we calculated the correlation of CaMKIIα phosphorilation with cortical spindle counts during the last 15 min before killing (**Fig 2A and 2B**). Spindle occurrence in the SWS and REM groups was assessed on spectral ratio maps (**Fig 2C and 2D**). While animals in the SWS group did not show any significant correlation (**Fig 2E** left panel), animals allowed to enter REM showed a positive correlation between spindle counts and pCaMKIIα levels (**Fig 2E** right panel). Interestingly, while pCaMKIIα levels were correlated to the sum of all spindles that occurred in the transition from SWS to REM (**Fig 2E** right panel, black dots and regression, R^2^ = 0.484, p = 0.0375), no correlations were observed for spindles occurring exclusively within SWS (**Fig 2E** right panels, blue dots and regression, R^2^ = 0.116, p = 0.369), nor for spindles sampled only from the IS state immediately before REM (**Fig 2E** right panels, magenta dots and regression, R^2^ = 0.007, p = 0.825).

**S3A Fig** shows that REM animals spent significantly more time in IS than SWS animals. REM animals did not have significantly more spindles than SWS animals (**S3B Fig**), but spindles lasted longer in IS than in SWS (**S3C Fig**). Altogether, more IS time in REM animals and longer spindles in IS explain why SWS animals displayed significantly less time with spindle occurrence (**S3D Fig**).

### Modeling Sleep-Dependent LTP in a Computational Network Fed with Hippocampal Spikes

The results above support the notion that LTP is triggered at the SWS/REM transition. To model the consequences of this phenomenon, we first investigated how state-dependent variations in firing regimes affect the synaptic weights of a fully-connected network comprising an excitatory population of stochastic binary units (see Materials and Methods). The synaptic weights were initialized with a random uniform distribution, and therefore with maximum entropy. A stable Hebbian learning rule based on pairwise synchrony was used to update synaptic weights over time [45]. Synchrony was evaluated in 4-ms bins, well within the interval of maximum STDP [47], and lack of synchrony led to a fixed amount of synaptic weight weakening. The simulations were generated by feeding the network with 2 kinds of external inputs (**S1 Table;** representative example): Poisson spike trains at various rates; and actual spike trains recorded from the rat hippocampal field CA1 during WK, SWS and REM [20].

**Fig 3:**
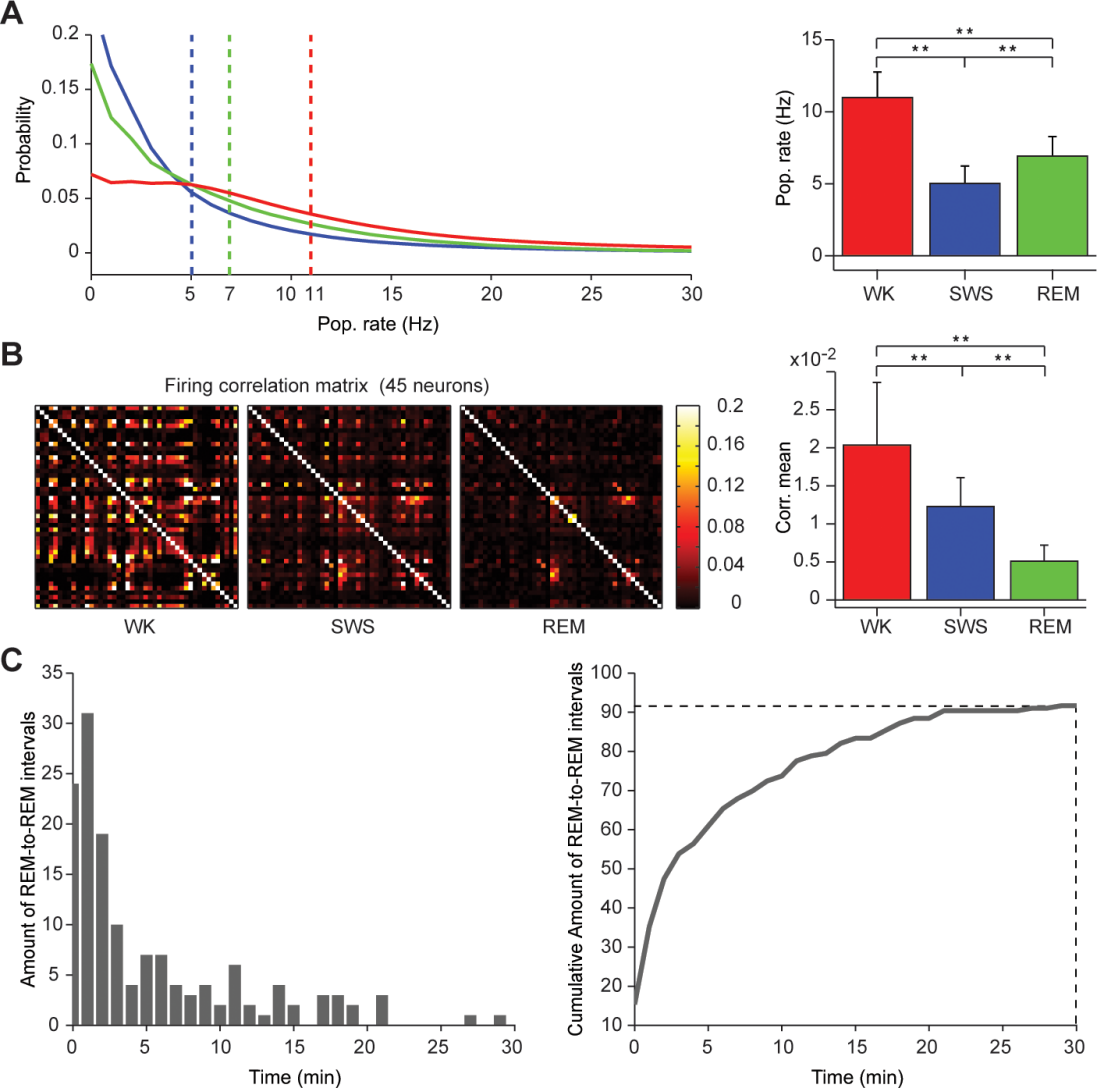
Statistics of real spikes from the hippocampal CA1 field during WK, SWS or REM. (A) Probability distributions of spike rates across states, with mean population values represented by dashed lines (left panel). Mean and variance of spike rates recorded during each state (right panel). (B) Square matrix of Pearson's linear correlation coefficient for spiking of 45 neurons during WK, SWS or REM (left panel), and the corresponding mean and variance (left panel). Significant differences of the Pearson's coefficient distribution were found between WK and SWS (Kolmogorov-Smirnov (KS) p = 6.9607e-023), WK and REM (KS, p = 1.0890e-023), and SWS and REM (p = 4.0259e-017). (C) Distribution of durations for intervals separating consecutive REM episodes (left panel, n = 6 rats; 28.8 hours of recordings). Cumulative plot of the REM-to-REM interval durations (right panel). Note that 91.6% of the intervals are shorter than 30 min (dashed line). More examples in **S4 Fig**; see also **S1 Table**.

The data statistics conformed to the expected state-dependent changes across the sleep-wake cycle [1–3]: SWS featured low mean firing rates (**Fig 3A**; representative example) and decreased firing synchronization (**Fig 3B**; representative example) compared with WK. REM was characterized by mean firing rates in between those of WK and SWS (**Fig 3A**), with very low firing synchronization (**Fig 3B**). For data from 5 other rats see **S4 Fig**. **Fig 3C** depicts the durations of intervals separating consecutive SWS/REM transitions (left panel; n = 6), and a cumulative plot showing that 91,6% of these intervals are shorter than 30 min (right panel).

Two major scenarios were implemented, with and without LTP during sleep. For statistical robustness, all simulations were independently repeated 25 times. The dynamics of the synaptic weight patterns were quantified using 2 metrics: the Similarity Index measured the sum of absolute differences between a given synaptic weight pattern and a reference pattern, while the Spearman´s correlation quantified ranking changes among synaptic weights compared to the reference pattern. Altogether, these metrics allowed us to estimate the rescaling and restructuring of the synaptic weight patterns over time. Rescaling was indicated by a reduction in the Similarity Index without major changes in Spearman´s correlation. Restructuring was indicated by a reduction in the Similarity Index accompanied by a reduction of Spearman´s correlation.

### Feeding the Network with Poisson Data

We began by characterizing the rate-dependency of synaptic weight dynamics during a regime of non-correlated external inputs. The network was fed Poisson surrogated spike trains with mean rates between 5 and 10 Hz, which represent the real data range (**Figs 3A and S4A**). The distribution of synaptic weights was rescaled over time to a narrow range of values (**S5A and S6A Figs**), exactly as observed previously for a single cell model [45], as well as a network model with both excitatory and inhibitory units [4]. The mean synaptic weight at the convergence time point (**S5A Fig**, dashed black lines) depended on the mean firing rate of the inputs **(S6B Fig**, blue curve). By the same token, the time required for the synaptic pattern to converge was inversely proportional to the input rate (**S6B Fig**, red curve).

For simulations with low rate inputs typical of SWS (**Figs 3 and S4A**), mean synaptic weights at the time of convergence were smaller than 0.5, resulting in net weakening (*M*_*w*_ *<0*, see Material and Methods) of the synaptic weights (**S5A and S6A Figs**, distributions for 3, 5 and 7Hz). However, synaptic weights that were initially below the mean at the time of convergence became strengthened (**S5A Fig**, bottom panels). Therefore, net weakening of synaptic weights does not imply that all synaptic weights decay over time, since weak synaptic connections are potentiated. Conversely, for simulations in which inputs had higher rates typical of REM or WK (>7Hz), synaptic weights at the time of convergence were larger than 0.5, resulting in net potentiation of synaptic weights (**S5A Fig,** for 10Hz and its corresponding gray distribution in **S5C and S6A Figs,** distributions for 12, 20 and 40Hz). Yet, synaptic weight values initially above the convergence range were reduced over time (**S5A Fig**, bottom panels). In summary, when the network was fed with non-correlated Poisson inputs, the wide range of synaptic weights used to initialize the network converged to a narrow and stable distribution, producing net weakening or net potentiation of the synapses for low and high firing rates, respectively.

### Feeding the Network with Real Spike Trains

To further characterize the state-dependency of synaptic weights, we fed the network with spike data from concatenated WK, SWS or REM episodes (**S5B Fig**). The simulations confirmed that the mean firing rates of the external inputs determine the mean value reached over time by the distribution of synaptic weights *P(w)*, as shown for Poisson data in previous work [45] and in the preceding section. Periods of increased spike rate and synchronization, such as WK, resulted in a smaller standard deviation of the synaptic weights when the network reached steady state, in comparison with periods of reduced spike rate and correlation, such as SWS or REM (**S1 Table** and **S5B and S5C Figs**green distributions). Note that the standard deviations at steady state were even smaller for non-correlated Poisson data of identical mean rates, and also obeyed the relationship WK<REM<SWS (**S1 Table** and **S5C Fig**, gray distributions).

**Fig 4:**
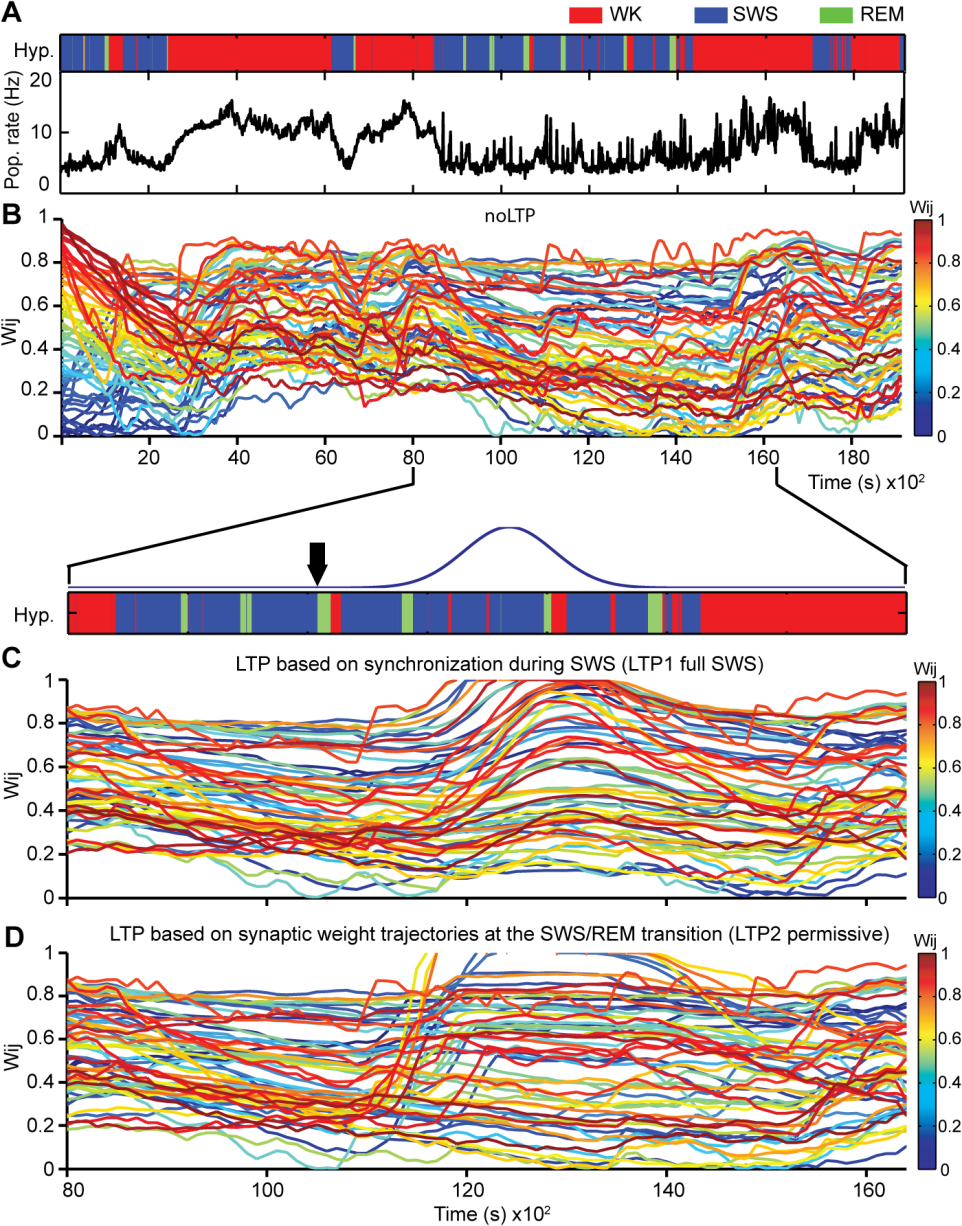
LTP during sleep leads to marked changes in synaptic weight trajectories. (A) Hypnogram of representative recording (top panel) and corresponding population spike rate activity over time (bottom panel). Three different simulations were run using real spike data: (B) without LTP; (C) with LTP 1 full SWS model, modulated by the amount of synchronized spikes observed during the SWS episode that preceded the 4^th^ REM period (duration of SWS episode = 658s), and (D) with LTP 2 permissive model, related to the changes in synaptic weight trajectories at the SWS/REM transition. Black arrow indicates the time point at the SWS/REM transition when the LTP Gaussian was triggered (~10,500s; 30s were elapsed for LTP evaluation before bonuses was applied). To focus on the sleep period, the (C) and (D) simulations are plotted from 8,000s to 16,200s. The initial synaptic weight values were uniformly distributed in the range [0..1]; consequently, colors (from dark blue to dark red) are also homogeneously distributed for w_*ij*_ values in [0..1]. Initial color maintained for each synaptic weight trajectory during entire simulation. In (B), bottom panel indicates the period during which the Gaussian curve affected the synaptic values to simulate LTP (blue curve). See also **S7 and S8 Figs.**

Next we investigated how external inputs with the real dynamics of state alternation affected the distribution of synaptic weights. **Fig 4**shows results when the network inputs were real spike data recorded over 5 hours from one rat (Rat1) cycling freely through the sleep-wake cycle, i.e. containing the natural alternation of WK, SWS and REM (**Fig 4A,** hypnogram**).** As expected, population firing rates were markedly state-dependent throughout the recording (**Fig 4A**, Pop. rate). The model displayed net potentiation during WK and net weakening during sleep (**Fig 4B**). We also observed that most synaptic connections did not reach extreme values (close to 0 or to 1) but converged to the middle range of possible values (**Fig 4B).**

### Modeling LTP during Sleep

Two alternative LTP models triggered near the SWS/REM transition were investigated. Since LTP is tightly associated with firing synchrony [11, 47, 54], one model attempted to capture the notion that SWS causes LTP through firing synchronization [55–57] and enhanced calcium influx [19, 30, 58], leading to calcium accumulation during SWS that would then lead to increased pCaMKIIα levels at REM onset. To simulate this scenario, LTP1 full SWS model applied a long-term bonus to each synapse starting at the SWS/REM transition, but according to the amount of pairwise synchrony observed throughout an entire SWS episode (87.06 ± 47.47, mean ±SEM in seconds, n = 6 rats).

The second model (LTP2) simulated short-term changes in pCaMKIIα levels as short-term increases in synaptic weights at the SWS/REM transition, and then used these short-term increases to determine long-term increases in synaptic weights. This model is compatible with the empirical data (**Figs 1 and 2**), and with the evidence of REM-dependent upregulation of plasticity factors [14–16, 20]. For each synaptic weight, LTP2 model coupled short-term changes at the SWS/REM transition to a gradual long-term bonus. The rationale for this coupling was the fact that the balance between high and low calcium influx in the millisecond scale is reflected in the balance between kinase and phosphatase activation in the seconds range, in particular CamKIIα, and determines the subsequent activation for over 30 min of Rho GTPases such as cdc42, and consequently to changes in gene regulation and protein synthesis in the hours scale [59–63].

To simulate this scenario, short-term changes in synaptic weights assessed for 60s at the transition from SWS (30s) to REM (30s) determined long-term bonuses applied for 30 min to the synaptic weights (see Material and Methods). Specifically, the angle formed by the synaptic weight trajectory at the transition from SWS to REM determined a long-term bonus. To comply with the notion that LTP requires positive calcium transients, LTP was applied exclusively to synaptic weights whose trajectory showed a positive slope during REM (LTP2 permissive 60s SWS/REM).

The long-term bonus applied in both models consisted of a Gaussian curve with onset at a reference SWS/REM boundary and peak value at 30 min, to match the duration of the spine-specific signaling cascade Ca^2+^–CaMKIIα–Cdc42 [64].

### LTP during Sleep May Lead to Pattern Restructuring

The evolution of synaptic weight patterns varied according to the LTP model employed. When LTP1 full SWS model was fed real data as inputs (**Fig 4C**), about 85% of the synapses underwent potentiation, in comparison with 21% in the No-LTP control. This resulted in a marked modulation of synaptic weight values (**S7D Fig,** 1^st^ and 2^nd^ panels), with a substantial net increase of the mean weight (**S7A Fig**, top panel, red curve) and increased spreading towards high synaptic weight values (**S7C Fig**, red with black edge distribution). Only 8% of the synapses underwent weakening, in comparison with 64% in the No-LTP control (**S6A Fig**, 1^st^ and 2^nd^ panels). The selection obeyed a uniform distribution across the synaptic weight range, so that even weak synaptic connections had a 16% chance of being potentiated. Overall, LTP model 1 led to a net potentiation of synaptic weights across their entire range (**S7D Fig**, middle panel), greatly exceeding that observed without LTP (**S7B Fig,** top panel, red curve). The distributions of synaptic weight changes (ΔW_ij_) showed a marked upward shift, with a concavity change on the quadratic fit of LTP1 full SWS model, in comparison with the fit for the No-LTP model (compare red and black curves in **S7E Fig**).

When LTP2 permissive model was fed real data (**Fig 4D**), about 48% of the synapses were recruited to undergo LTP (**S7D Fig,** right panel, red bar), substantially less than in the case of LTP1 full SWS model. LTP2 permissive model also led to net synaptic potentiation across the entire range of weights, but many more connections were de-potentiated in comparison with LTP1 full SWS model (compare with middle panel). The distributions of differences between final and initial synaptic weight values (**S7E Fig**, blue) show an upward shift without concavity change in which most changes affect weak connections, i.e. the shift was largest for the lowest initial synaptic weights, and decreased gradually for larger initial weights.

**Fig 5:**
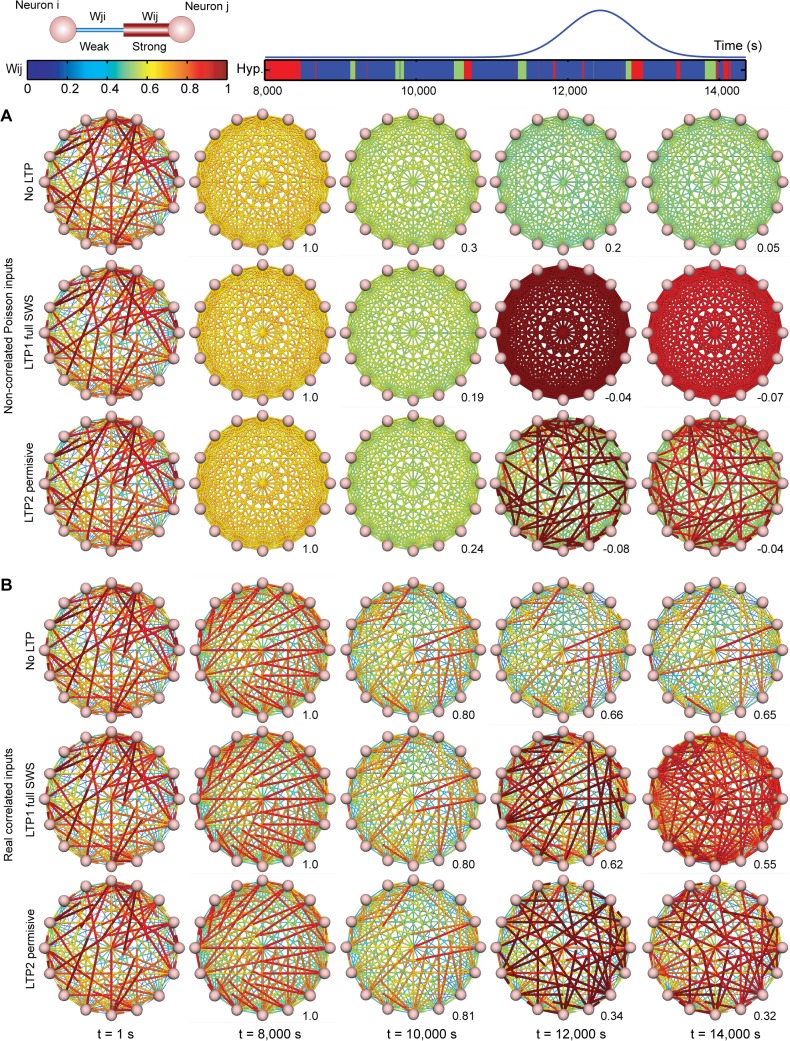
Synaptic patterns are downscaled during sleep without LTP, but undergo restructuring after sleep-dependent LTP. Fully connected network of 16 neurons with synapses (cylinders); strength indicated by cylinder radius and colors spanning blue for weak, red for strong. For comparison, all simulations were initialized with the same random uniform distribution of synaptic weights (column t = 1s). (A) Simulations with Poisson inputs. (B) Simulations with real inputs. The rows in each panel represent the No LTP model, LTP1 full SWS model and LTP2 permissive model (top to bottom). The columns represent 5 simulation time-points; Spearman´s correlation values (bottom right) with reference pattern t = 8,000s.

The different consequences of the LTP models (LTP1 full SWS and LTP2 permissive) are shown in **Fig 5**, which depicts the temporal evolution of a fully connected network of 16 neurons under Poisson (**Fig 5A**) or real data regimes (**Fig 5B**). The initial synaptic weight pattern (**Fig 5A**, 1^st^ column) was quickly erased when Poisson-distributed data were used as inputs (**Fig 5A**, 2^nd^ and 3^rd^ columns). LTP1 full SWS model uniformly enhanced all synaptic weights, while LTP2 permissive model embossed a new pattern (**Fig 5A**, 4^th^ and 5^th^ columns).

Real data allowed for a much longer persistence of the initial pattern, which evolved in distinct ways for LTP1 full SWS and LTP2 permissive. While the pattern changed monotonically over time in LTP1 full SWS model (**Fig 5B**, 2^nd^ row), LTP2 restrictive model caused a major revamp of synaptic weights (**Fig 5B**, 3^rd^ row), so that the pattern that emerged at the end of the simulation was very different from the initial pattern.

**Fig 6:**
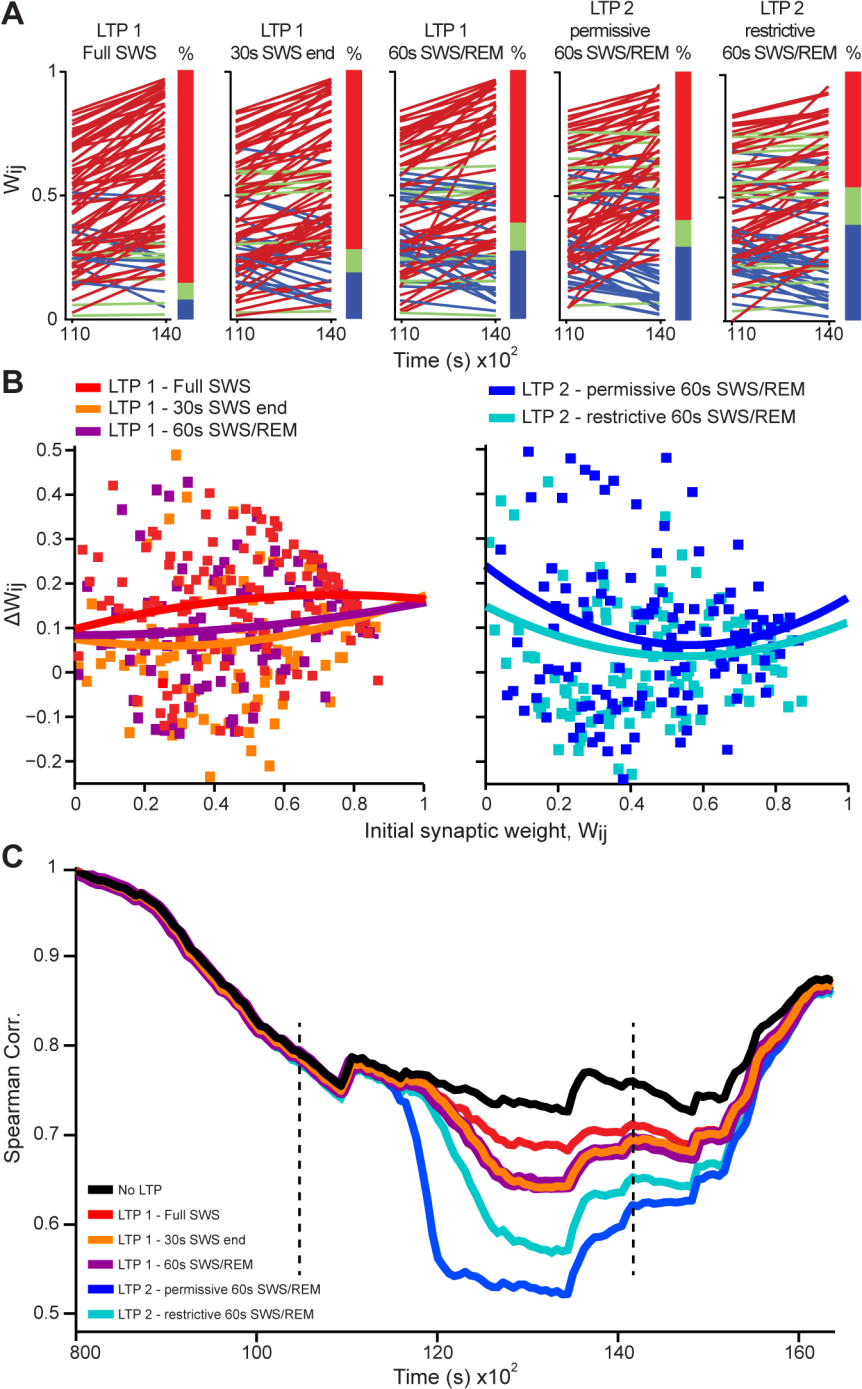
LTP model variations show that evaluation around the SWS/REM transition leads to pattern restructuring. (A) Initial and final synaptic weight values across the time interval of LTP simulation (11,000–14,000s) for variations of the LTP 1 and LTP 2 models. Red, green and blue lines represent positive, near-zero and negative slopes, with corresponding percentages indicated by the color bar. 1^st^ panel for LTP 1 model evaluated over an entire SWS episode; 2^nd^ panel for LTP 1 model evaluated only during the last 30s of SWS immediately before REM; 3^rd^ panel for LTP 1 model evaluated using the last 30s SWS period plus the following 30s of REM; 4^th^ panel for LTP 2 model using all positive REM slopes (permissive); 5^th^ panel for LTP 2 model using only positive REM slopes that were also larger than the SWS slope (restrictive). For details of restrictive and permissive LTP 2 models, see **S10 Fig**. (B) Initial synaptic weight values (x axis) versus difference between initial and last time-points of LTP simulation (ΔWij, y axis). The curves are quadratic fits. Left and right panels for the variations of LTP 1 and LTP 2 models, respectively. (C) Spearman´s correlations over time for variations of LTP 1 and LTP 2 models, comparing the distribution of w_*ij*_(t) values at the time *t* with the initial distribution of w_*ij*_(0). Dashed lines indicate the time range [10,500s to 14,100s] when the LTP Gaussian was applied (boundaries of LTP simulation).

Were the differences between LTP1 and LTP2 models related to the different durations of the inputs, to the role assigned to the SWS/REM transition, or to intrinsic mechanistic differences between the models? To address these questions, we futher simulated LTP1 using not an entire SWS episode, but either a limited 30s SWS period immediately before REM (LTP1 - 30s SWS end), or that 30s SWS period plus the following 30s of REM (LTP1 - 60s SWS/REM). We found that short sleep periods near the SWS/REM transition produced less net synaptic potentiation for LTP1 than full SWS episodes **(Fig 6A and 6B)**. They also produced more pattern restructuring than full SWS episodes (i.e. decrease in Spearman´s correlations, **Fig 6C**), partially replicating the results of LTP2 permissive model. Next we compared LTP2 permissive model with an alternative in which LTP2 was applied only when the REM slope was positive and larger than the SWS slope (LTP2 restrictive) (see Material and Methods). This more restrictive version of LTP2 led to less potentiation (**Fig 6A,** last two panels; **Fig 6B**, right panel), and less pattern restructuring **(Fig 6C),** than the original, more permissive LTP2.

**Fig 7:**
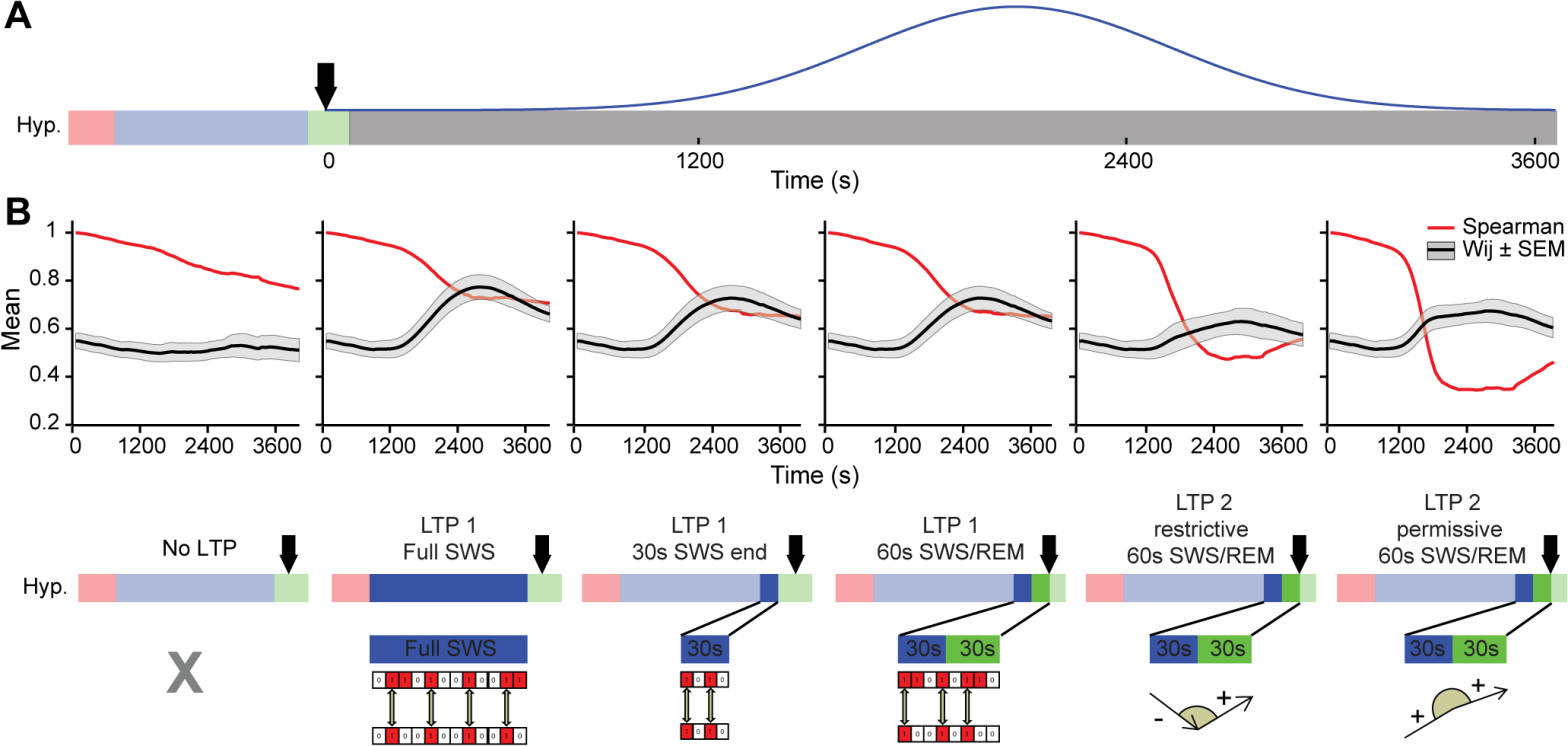
Group data (n = 6) show pattern restructuring when LTP is evaluated near the SWS/REM transition. (A) LTP Gaussian triggered near the SWS/REM transition. (B) Mean Spearman´s correlations (red lines) and mean synaptic weight values (black lines ±SEM as shaded area) during the time period of Gaussian curve application (~3,600s). Note the quasi-monotonic decay of Spearman’s correlations from left to right panels, corresponding to the 5 LTP models studied (bottom row); See S10 Fig.

Of note, the case presented so far is representative of 5 other animals, despite the high variability of the spike datasets used as inputs, which resulted from traversing quite different real sleep-wake cycles (**S8 Fig**). Spearman´s correlations and mean synaptic weights **(Fig 7)**, used respectively to characterize restructuring and rescaling, confirm across animals that LTP during sleep leads to pattern restructuring, especially when assessed at the SWS/REM transition.

### Synaptic Rescaling Favors Synaptic Restructuring

**Fig 8:**
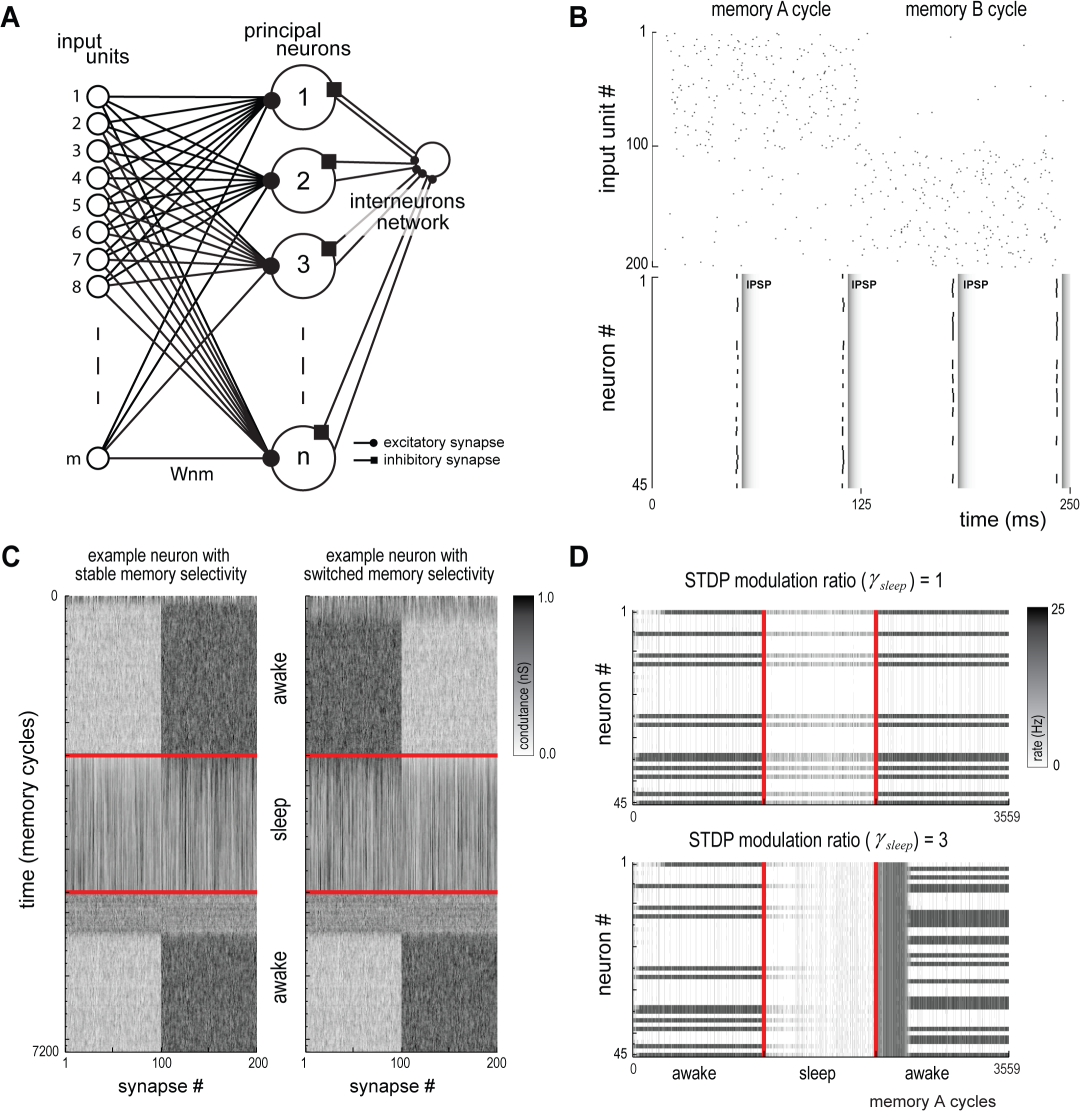
Canonical hippocampal-cortical model. (A) Network model with an input layer, a principal neurons layer and an interneurons layer. (B) Input spikes shown for two different input cycles (top). Alternated memories are active in each cycle. Cells #1 to #100 belong to memory A and cells #101 to #200 belong to memory B. Postsynaptic spikes and Inhibitory Postsynaptic Potentials (IPSP) release shown following the above input (bottom). (C) Conductance of all synapses of two representative neurons during a full simulation run: one neuron whose memory selectivity remains stable after a sleep cycle (left); and one neuron whose memory selectivity is switched following the sleep cycle (right). (D) Population activity for all cycles with a specific memory on shown for two conditions of STDP modulation: (top) no *C*_*p*_ */C*_*d*_ modulation during sleep leads to no change in the response pattern of the population; (bottom) high *C*_*p*_ */C*_*d*_ modulation leads to complete restructuring of the response pattern of the population.

Since both SHY and the synaptic embossing hypothesis have empirical support, it is possible that the use of both mechanisms is synergistic. To clarify this point, we used a network model based on a canonical hippocampal-cortical circuit capable of developing specificity in response to concurrent inputs [50–52] (Fig 8). The model was adapted by implementing plasticity mechanisms analogous to SHY and the synaptic embossing hypothesis (see Material and Methods). The effect of sleep on memory was studied by comparing the patterns of synaptic weights post-sleep with pre-sleep (to assess the level of memory restructuring) and with the pattern reinforced by sleep-dependent LTP (to assess the influence of the SWS/REM transition pattern over the established memory).

**Fig 9:**
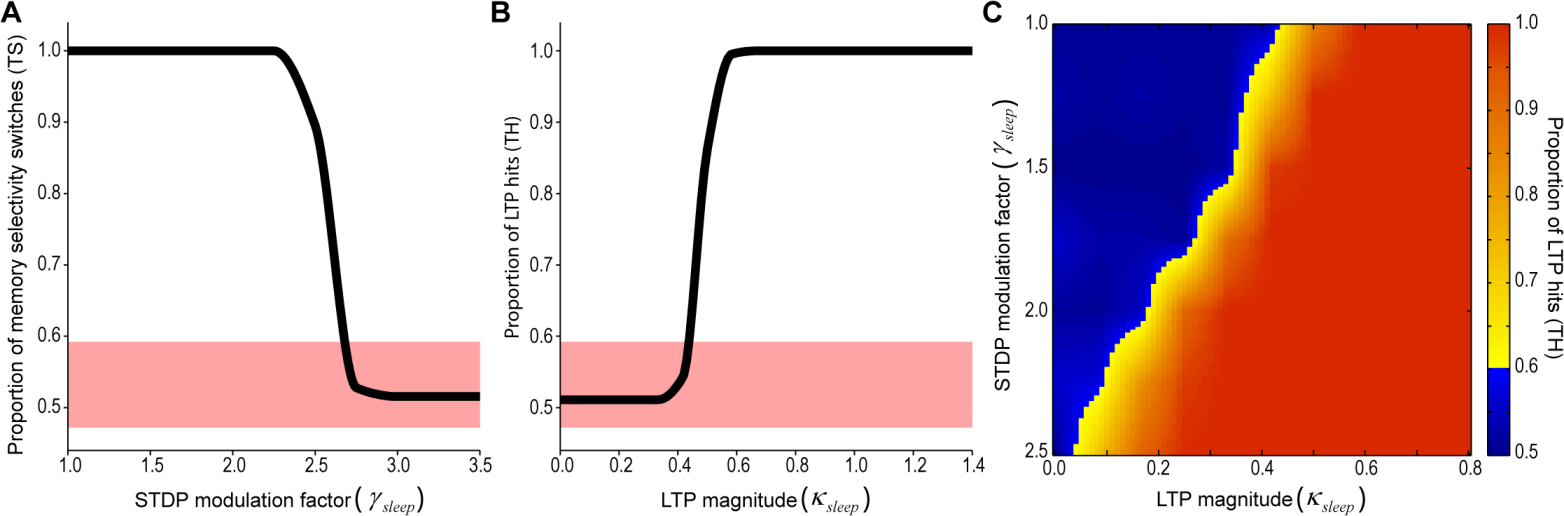
Functional relationship between the synaptic homeostasis and embossing hypotheses. (A) Proportion of neurons with stable memory selectivity after sleep (T_*S*_) as a function of STDP modulation factor (*γ*_*sleep*_) (mean, n = 50). Colored interval denotes the range by which the measure is not significantly (p>0.05) different from random trials. (B) Proportion of LTP hits (T_*H*_) as a function of overall LTP gain (*κ*_*sleep*_) (mean, n = 50). Colored interval denotes the range in which the measure is not significantly (p>0.05) different from random trials. (C) Color coded plot of previous metric as a function of both STDP modulation factor (*γ*_*sleep*_) and overall LTP gain (*κ*_*sleep*_). Yellow to red shades are values with significant number of memory hits. Blue shades cannot be distinguished from random runs.

Synaptic homeostasis was implemented by modulating the STDP rule [47, 54] during sleep through weakening of potentiation and strengthening of depression [4]. As observed in our empirical data, the rate of the input units was reduced during sleep. The effect of sleep on synaptic restructuring was measured by assessing the number of neurons whose pattern selectivity was either stable or switched. We observed that an increase in STDP modulation led to a number of stable memories, as expected by a random permutation of the pattern selectivity (**Fig 9A**). Although this experiment demonstrates that synaptic homeostasis shuffles synaptic weights, there is no mechanism to determine the output synaptic structure, i.e., SHY allows an acquired memory to be erased, but it cannot promote a specific pattern into the synaptic weights.

Embossing was implemented by evoking LTP on selected synapses during sleep. The LTP pattern was set proportional to the synaptic weights of another neuron selected randomly at a time previous to sleep onset. LTP control over the synaptic organization could be measured by comparing the memory of the neuron after sleep with the memory of the neuron selected as reference for LTP just before sleep onset. We observed that an increase of the intensity of LTP modulation led to a full control of the memory by the evoked LTP (**Fig 9B**).

We then implemented both hypotheses and observed the development of synaptic reorganization in the model. We simulated sleep cycles for a range of values of STDP modulation and LTP intensity and observed the number of memory hits between the pattern following the sleep cycle and the reference pattern with evoked LTP (**Fig 9C**). With an increasing level of STDP modulation, a lower LTP intensity was required to enhance LTP control, although some level of LTP was still needed to ensure LTP control over the synaptic organization. This result indicates that synaptic homeostasis facilitates controlled synaptic restructuring during synaptic embossing.

## Discussion

The experience-dependent increase of pCaMKIIα levels in hippocampal cell layers from SWS to REM indicates that the latter triggers synaptic potentiation within circuits selected by waking experience. Our data also show that CaMKIIα phosphorylation during sleep was proportional not to slow-wave or theta oscillations, respectively markers of SWS or REM [39], but rather to the amount of cortical spindles that occur at the SWS/REM transition. Spindles are 7–14 Hz oscillations that occur during sleep [39, 42], are abundant at the transition from SWS to REM [43], gate hippocampo-cortical communication [65], and correlate strongly with sleep-dependent learning [66–68]. Cortical spindles have been proposed to play an important role in calcium-dependent plasticity [30], perhaps by coupling the changes in memory traces produced by SWS to REM-triggered gene expression required for long-term storage [20].

The tight relationship between spindle counts and hippocampal pCaMKIIα levels likely reflects the key role played by spindles in firing synchronization across the hippocampo-cortical axis [65, 69]. Neither SWS or IS alone showed correlation between spindle count and pCaMKIIα levels, which suggests that cortical spindles spanning SWS and IS are critical for the coupling of the neuronal changes produced during SWS to hippocampal CaMKIIα phosphorylation at REM onset. A similar relationship between LFP power in the spindle range and Zif-268 mRNA expression 30 min after REM [20] supports a causal chain linking spindles, CaMKIIα phosphorylation and Zif-268 induction. Non-REM sleep has recently been implicated with learning-related formation of dendritic spines [21], but REM deprivation in this study was only partial (about 80% of the total), and spared SWS/REM transitions. Determination of the precise role of these transitions for sleep-dependent plasticity requires further investigation. Overall, our results are compatible with the notion that sleep spindles “open the molecular gates to plasticity” [30].

Based on the empirical data, we simulated a homeostatic excitatory network fed with spike data from the hippocampus of behaving rats to compare alternative theories regarding plasticity during sleep. In the 1^st^ scenario, which did not include sleep-dependent LTP and was compatible with SHY, synaptic weights inexorably converged to the center of the distribution. In the 2^nd^ scenario, which included sleep-dependent LTP, synaptic weights showed marked changes compatible with either orderly synaptic up-scaling (for synchronization-based LTP computed over entire SWS episodes) or with disorderly synaptic embossing (for LTP triggered at the SWS/REM transition).

### Rescaling in the Absence of LTP during Sleep

Rescaling occurred in the absence of LTP during sleep, and was most pronounced when non-correlated Poisson inputs were used (**Figs 4A and S4A**). Synaptic weights quickly converged to a narrow range of values as previously described for different networks that reach an equilibrium state through a homeostatic process [7], including a network with inhibitory inputs [4]. Net weakening or potentiation depended on how low or high was the external activity. Non-correlated Poisson activity with high rate caused a faster decrease in the diversity of synaptic weight values than correlated spiking at lower rates (**S4A Fig**). Similarly, REM-only inputs were more effective in rescaling than SWS-only inputs (**S4B and S4C Fig**), which contradicts SHY [7] but agrees with the empirical findings of decreased firing rates after REM [25].

When the network received correlated real data inputs, the resulting synaptic weight distributions were much wider but still converged to the center (**S4B Fig**), with a tight relationship between input rates and the mean synaptic weight at the end of the simulation. These results conform to the notion that a network that goes through sleep without LTP remains stable, avoiding extremely weak or strong synaptic weights [45]. Synaptic weight convergence was orderly, preserving synaptic weight ranks as indicated by the relatively stable values of Spearman´s correlations over time (**S6A Fig**, bottom panel, black curve, 10,000s to 14,500s interval). While synaptic weight ranks were preserved in the absence of LTP, mean synaptic weights progressively decreased (**S6A Fig**, top panel, black curve), reaching the lowest value during sleep. The same occurred for the Similarity Index (**S6A Fig**, middle panel, black curve). Without LTP during sleep, net synaptic weights were downscaled, and the initial pattern was rescaled.

### Restructuring Due to Sleep-Dependent LTP

When LTP was modulated by the spike synchrony exhibited during an entire SWS episode, synapses were uniformly selected for potentiation (LTP1 full SWS model). When synapses were potentiated based on the angle formed by the synaptic weight trajectories at the SWS/REM transition (LTP2 model), synaptic recruitment was also quite uniform but the magnitude of potentiation was stronger for weights that were initially low (**S6E Fig**, blue curve). In both models, synaptic weight values were scattered away from the convergence range (**Figs 4C, 4D**and **S6C** middle, right panel, distribution with black edges).

Real neuronal activity imposes network relations that do not exist for Poisson inputs, including inhomogeneous firing rate variability and synchronous firing among neurons. These conditions determine that specific connections undergo markedly different dynamics under LTP1 and LTP2 models. The most interesting cases are those in which synaptic weights undergo opposite changes. About 85% of the connections undergo potentiation under LTP1 full SWS model but no modulation or weakening under LTP2 permissive model (**S6E Fig**, triangles). In contrast, only 8% of the connections undergo no modulation or weakening under LTP1 model, but show potentiation under LTP2 model (**S6E Fig**, squares). Interestingly, the results produced by LTP1 and LTP2 models converged when the former was evaluated around the SWS/REM transition; or when the latter was more restrictive (**Fig 6**).

The results support the notion that LTP during sleep allows weaker synaptic connections to also play a role in mnemonic processing [70]. Weight-dependent plasticity of hippocampal neurons, favoring weak over strong synapses, has been shown *in vitro* [45, 47]. Here, the combination of real hippocampal inputs and LTP during sleep triggered long-term changes in the synaptic weights that were specific of the particular LTP mechanism simulated. The consequences of LTP1 model during sleep were overall synaptic potentiation, increased similarity with the initial pattern and preservation of synaptic weight ranks. For LTP1 and LTP2 models evaluated at the SWS/REM transition, sleep gave rise to new synaptic weight patterns, as indicated the low Spearman´s correlation values (**Fig 5B**, last row, from 10,000s to 14,000s). Given that the typical period of the sleep cycle in rats is below 2 min (**Fig 3C,** left panel), with 91,6% of the episodes under the 30 min LTP Gaussian peak (**Fig 3C,** right panel), the sleep-dependent synaptic weight changes introduced by LTP models are prone to stagger from cycle to cycle, leading to progressively different patterns over time.

SHY does not seem to account for all the cognitive effects of sleep, which not only protects memories passively from retroactive interference, but can actively enhance specific memories in detriment of others, and lead to novel insights [71–79]. A simplified integrate-and-fire SHY model exclusively composed of excitatory neurons was recently used to simulate gist extraction and integration of new with old memories without the need of LTP during sleep [10,80]. These properties derive directly from the down-selection of weak synapses and protection of strong synapses, which end up eliminating weak memories (supposedly “spurious” information) and therefore increasing the signal-to-noise ratio of memory retrieval. However, by the same token, such mechanisms are not bound to explain the additional information content that arises from memory restructuring during sleep [71–79]. For instance, a recent study of perceptual learning in humans found that REM rescues memories from interference, preferentially strengthening weak memories [81].

In contrast, the results presented here for Model 1 show that LTP calculated over short periods near the SWS/REM transition lead to pattern restructuring compatible with the synaptic embossing theory. Furthermore, the more realistic hippocampal-cortical architecture with both excitatory and inhibitory synapses (Model 2) showed that the restructuring of synaptic patterns due to LTP was enhanced by homeostasis, which could explain the sleep-dependent enhancement of specific memories. These results highlight the importance of the SWS/REM coupling, providing strong support for the sequential hypothesis of mnemonic processing during sleep [6, 12, 27]. The joint occurrence of Hebbian and non-Hebbian plasticity during sleep, which leads to new synaptic patterns embossed over a background of homeostatically rescaled synaptic weights, may ultimately explain the positive role of sleep in the cognition.

## Supporting Information

S1 Text Implants and electrophysiological recordings. (DOCX)

S2 Text Sorting of sleep-wake states with spectral maps and behavior. (DOCX)

S3 Text Immunohistochemistry. (DOCX)

S4 Text Quantification of protein levels. (DOCX)

S5 Text Electrophysiological quantification. (DOCX)

S6 Text Canonical hippocampal-cortical network (Model 2). (DOCX)

S1 Fig Experimental design. All animals showed intense exploratory activity during exposure to the novel objects (40–90% of the time spent in active exploration, mean 70%). Following novel object exploration (A) or not (B), rats were kept awake for 3 hours and were then allowed to sleep. After one criterion episode of WK, SWS or REM, rats were immediately killed, the brains were frozen and processed for immunohistochemistry. IS represents the intermediate sleep state that separates SWS from REM [43]. Drawings adapted from [20].
(TIF)

S2 Fig Immunohistochemical labeling patterns and antibody specificity. (A) Labeling patterns in the dorsal CA1 field of the hippocampus for pCaMKIIα, total CaMKIIα, Zif-268 and Actin. The anti-pCaMKIIα antibody led to cytoplasmic labeling, marking both the soma and the neuropil (arrows in top center panels). The anti-Zif-268 antibody produced nuclear labeling (arrows in bottom center panels). (B) Antibody specificity was confirmed in immunoblots using hippocampus extracts.
(TIF)

S3 Fig Statistical features of IS and spindles in the SWS and REM groups. (A) REM animals spent significantly more time in IS than SWS animals. (B) REM animals did not show a significantly greater count of spindles than SWS animals. (C) In the REM group, spindles lasted significantly longer in IS than in SWS. (D) Overall, animals in the REM group spent significantly more time with spindle activity than animals in the SWS group. All results for data collected from the parietal lead (* p < 0.05, *** p < 0.01, unpaired t test).
(TIF)

S4 Fig Statistical features of real spike data recorded from 5 other rats. (A) Spike rates and (B) Pearson's linear correlation mean and variance for spiking during WK, SWS or REM. Data recorded from the CA1 field of the hippocampus of 5 rats (columns A2—A6).
(TIF)

S5 Fig Without sleep-dependent LTP, both Poisson and real spike inputs lead to fast, rate-proportional, convergent rescaling of network synaptic weights. (A) Synaptic weight variation over time (*w(t)*) when the network is exposed to Poisson inputs at 5Hz, 7Hz, or 10Hz (columns). First row: colored areas represent the dynamics of the synaptic weight values. Second row: synaptic weight trajectories over time allow for a better visualization of the convergence points in the vertical axes. (B) Simulations run for separate sleep states. Real episodes of WK, SWS and REM were concatenated, and their corresponding spike activity was used to feed the network. Dynamics of the synaptic weight values in the first column, synaptic weight trajectories in the second column. In (A) and (B) simulations, the initial synaptic weight values were uniformly distributed in the range [0…1], and the colors attributed for each *w*_*ij*_ values change over time, reflecting synaptic weight changes. (C) Final synaptic weight distributions (green area) obtained at the convergence time point (dash black line in panel B) for each stage (WK, SWS and REM, from left to right panels). The corresponding final distributions for Poisson inputs are also displayed (gray area, same mean firing rate as in panel A).
(TIF)

S6 Fig Input rates are proportional to final mean weights and inversely proportional to time of convergence. (A) Weight distributions at the convergence time point for every Poisson inputs rate simulated. Firing rates ≤ 7Hz or >7Hz caused net synaptic downscaling (Mw<0) or up-scaling (Mw>0), respectively. (B) As external rates increase, convergence time decreases exponentially (red), and the mean weight achieved at the time of convergence increases logarithmically (blue).
(TIF)

S7 Fig Evolution of synaptic weights with and without LTP during sleep. (A) Top LTP1 full SWS model panel shows mean synaptic weights for simulations without LTP (black), with synchronization-based LTP during SWS (red), and with LTP based on synaptic trajectories at the SWS/REM transition (blue). The middle panels show the similarity index over time, comparing the current distribution of w_*ij*_(t) values at the time *t* with the initial distribution of *w*_*ij*_(0). The bottom panel shows Spearman´s correlations over time, comparing the distribution of w_*ij*_(t) with w_*ij*_(0). (B) Subtractions of mean weights (ΔMean) between LTP and non-LTP models. For A and B panels, dashed lines indicate the time range [10,500s to 14,100s] during which the LTP Gaussian was applied, i.e. boundaries of LTP simulation. (C) Synaptic weight distributions before (9,200s, light colors) and after LTP during sleep (14,000s, dark colors). (D) Changes in synaptic weight values for the initial (11,000s) and last (14,000s) time-points of LTP simulation. Red, green and blue lines represent positive, near-zero and negative slopes, with corresponding percentages indicated by the color bar on the right. (E) Initial synaptic weight values (x axis) versus difference between initial and last time-points of LTP simulation (ΔWij, y axis). Compare the results of the non-LTP model (black) with LTP models 1 and 2 (red and blue, respectively). The curves are quadratic fits. Less of the weak synaptic connections were selected for potentiation in LTP model 2 than in the case of LTP model 1 (10% and 16%, respectively). Notwithstanding, the former were more potentiated overall (**S7E Fig**, blue curve). There was a net increase in mean synaptic weight value (**S7A Fig**, top panel, blue curve), and increased spreading towards high synaptic weight values, but with a preservation of low synaptic weights as well (**S7C Fig**, blue with black edge distribution). A total of 36% of the synapses underwent down-scaling (**S7D Fig,** right panel, blue bar). Net up-scaling of synaptic weights also occurred for LTP model 2 (**S7A and S7B Fig**, top panels, blue curves), although with a lesser magnitude than for LTP model 1 (compare red and blue curves in **S7A Fig**). A subtraction of mean weights (ΔMean) between the two models shows that weights under LTP model 2 rise faster but not as much as in the case of LTP model 1, so the initial positive difference is soon followed by a negative ΔMean deflection (**S7B Fig**, top panel, green curve). LTP model 1 during SWS led to an increase in the similarity between the initial synaptic weight pattern and the patterns evolving over time, in comparison with non-LTP simulations. This occurred because LTP restored synaptic weights that would have been downscaled without LTP, bringing them closer to the initial conditions (**S7A and S7B Fig**, top panels). On the other hand, Spearman’s correlations were very similar between LTP model 1 and non-LTP simulations (**S7A Fig,** bottom panel, compare the red and black curves). This indicates that the synaptic up-scaling promoted by synchronization-based LTP is highly orderly, and does not lead to changes in synaptic weight ranking, i.e. does not cause pattern restructuring. The dynamics of the Spearman´s correlation when patterns with and without LTP model 1 are compared shows a stable trajectory near 1.0, indicating high-fidelity preservation of synaptic weight ranks (**S7B Fig**, bottom panel, red trace). A very different picture emerged for LTP model 2. While the similarity between the initial synaptic weight pattern and the patterns evolving over time was not markedly different among LTP models (**S7A Fig**, middle panel), LTP model 2 caused a major redistribution of synaptic weights, indicated by a marked decrease of Spearman’s correlations (**S7A Fig**, bottom panel), with a corresponding increase of the synaptic weight span (**S7C Fig**, blue distributions).S8 Fig Hypnograms of additional 5 rats, with indication of LTP onset.
(TIF)

S8 Fig Hypnograms of additional 5 rats, with indication of LTP onset. LTP Gaussian curve profiles over real sleep-wake cycles (hypnograms). Black arrows indicates the LTP onsets; n = 5 rats, rows Rat2—Rat6.
(TIF)

S9 Fig Example of spontaneous network activity (Model 1). (A) Spike rastergram and its corresponding average population firing rate (network size = 100 neurons). (B) Adjusted sigmoid function used as the probability (P) to update neuron state (see Material and Methods)
(TIF)

S10 Fig Ways to calculate *Cieg*_*ij*_ for simulations with LTP (Model 1). (A) LTP1 was based on spike synchronization during SWS. We counted the spikes when the pre (j) and post (i) synaptic neurons fired simultaneously (represented by *Sp*_*ij*_) over the amount of spikes of the pre-synaptic neuron j (represented by *Sp*_*j*_). This was calculated during a period of SWS previous to the REM stage. (B) In LTP2, *Cieg*_*ij*_ varied linearly according to the angle β formed by the *W*_*ij*_ *(t)* values at the SWS➔REM transition. The left panel shows a contingency table of the possible changes in synaptic trajectory slopes at the SWS➔REM transition. The right panel shows two variations of the model, based on different relations between β and *Cieg*. The most permissive model (dark blue line) applied LTP to all positive slopes during REM, irrespective of the SWS slope (cases 1, 2 and 3). The more restrictive model (light blue) applied LTP only when the REM slope was both positive and larger than the SWS slope (β<π; cases 1 and 2 but not 3). (C) Schematic representation of LTP2, showing the relationship between short-term changes in synaptic trajectory at the SWS➔REM transition and the long-term changes in synaptic weights (LTP), which followed a Gaussian curve triggered at time t_T_ with peak at t_T_+ 1,800s.
(TIF)

S1 Table External inputs and synaptic weights at convergence time points for rat A1. (XLSX)
