# Supplementary material for: Synaptic Homeostasis and Restructuring across the Sleep-Wake Cycle
Source: PLoS Comput Biol. 2015 May 28;11(5):e1004241. doi: 10.1371/journal.pcbi.1004241 (PMC4447375; doi:10.1371/journal.pcbi.1004241)
Supplement: S1 Text — (DOCX) [file pcbi.1004241.s001.docx]

## Text S1. Implants and electrophysiological recordings.

Animals were implanted with monopolar LFP electrodes (stainless steel microscrews) for cortical and reference leads, and Teflon-coated stainless steel microwires (125 μm diameter; A-M Systems, USA) for the dentate gyrus (DG). Animals pretreated with pentabiotics (19 mg/kg) received ketamine (100 mg/kg i.m.), xylazine (40 mg/kg i.m.) and atropine (0.05 ml s.c.), and were positioned in a stereotaxic frame (David Kopf Instruments, USA). Cortical leads were positioned in the parietal bone (AP = -1.0 mm, -3.0 mm, and -5.0 mm; ML = 2.5 mm from Bregma); reference lead in the nasal bone. DG coordinates were AP = -2.8 mm, ML = 1.5 mm from Bregma, DV = -3.3 mm from the pia [Paxinos G, Watson C (1998) The rat brain in stereotaxic coordinates. San Diego: Academic Press]. Electrodes were soldered to a miniature connector (Omnetics, USA) and fixed onto the skull with dental acrylic. After surgery, rats were individually housed on a 12:12h light/dark schedule, lights on at 06:00, food and water ad libitum. After 3 days for recovery, animals were handled for 3 days to decrease stress. LFPs were recorded with a 32-channel multineuron acquisition processor (Plexon, USA) using 1000X amplification, filtering 0.7 – 170 Hz, and digitalization at 500 Hz. Video recordings and animal tracking were performed with a CinePlex system (Plexon, USA) that synchronized neural and behavioral data.
