# Supplementary material for: Synaptic Homeostasis and Restructuring across the Sleep-Wake Cycle
Source: PLoS Comput Biol. 2015 May 28;11(5):e1004241. doi: 10.1371/journal.pcbi.1004241 (PMC4447375; doi:10.1371/journal.pcbi.1004241)
Supplement: S2 Text — (DOCX) [file pcbi.1004241.s002.docx]

## Text S2. Sorting of sleep-wake states with spectral maps and behavior.

The axes of the spectral map were two spectral ratios, calculated by dividing integrated LFP spectral amplitudes at selected frequency bands: (0.5–20)/(0.5–55) Hz for abscissa (Spectral ratio 1) and (0.5–4.5)/(0.5–9) Hz for ordinate (Spectral ratio 2). The routine was used to confirm group assignment, and analyze spindle activity. Major sleep-wake cycle states were identified: WK (active exploration with sustained whisking and theta rhythm), SWS (body stillness, eyes closed and large-amplitude slow oscillations), and REM (body stillness with intermittent whisking, eyes closed and theta rhythm).
