# Supplementary material for: Synaptic Homeostasis and Restructuring across the Sleep-Wake Cycle
Source: PLoS Comput Biol. 2015 May 28;11(5):e1004241. doi: 10.1371/journal.pcbi.1004241 (PMC4447375; doi:10.1371/journal.pcbi.1004241)
Supplement: S3 Text — (DOCX) [file pcbi.1004241.s003.docx]

## Text S3. Immunohistochemistry.

Rats were anesthetized with isoflurane 5%, and decapitated. Brains were quickly removed, and stored at -80^o^C. The left hemisphere was frontally sectioned at 20 μm in a cryostat (Micron HM 550, Germany), thaw-mounted on glass slides (SuperFrost Plus, VWR International, USA), and post-fixed on 4% paraformaldehyde. For histological confirmation, serial sections were stained with 0.1% cresyl violet. The remaining sections were washed for 20 min in 0.1 M phosphate buffer pH 7.4 (PB) and incubated in a blocking buffer solution (0.5% fresh skim milk and 0.3% Triton X-100 in 0.1 M PB) for 30 min. Sections were then incubated overnight at 18^o^C in rabbit polyclonal antibody anti-phospho-CaMKIIα (Thr286) (Millipore catalog # AB3827, 1:200 in blocking buffer), mouse monoclonal total CaMKIIα (Santa Cruz Biotechnology catalog # sc-13141, 1:50 in blocking buffer), mouse monoclonal Actin (Sigma catalog #A3853, 1:180 in blocking buffer) or rabbit polyclonal antibody anti-Egr-1 (Zif-268, C-19, Santa Cruz Biotechnology catalog # sc-189, 1:200 in blocking buffer), washed in PB (2x, 10 min each), incubated with biotinylated goat anti-rabbit or anti-mouse IgG (1:200 in blocking buffer, Vector Labs, USA) for 2 h, washed in PB (2x, 10 min each), and then incubated for 2 h in avidin-biotin-peroxidase solution (Vector Labs, USA). Slides were placed in a solution containing 0.03% DAB and 0.001% hydrogen peroxide in 0.1 M PB, dehydrated and cover-slipped. To determine labeling specificity, the primary antibody was replaced by blocking buffer in test sections. Labeling specificity for pCaMKIIα, total CaMKIIα, Actin, and Zif-268 (**Figure S2A**) was also confirmed by Western blots of brain extracts (**Figure S2B**) prepared in buffer containing 20 mM Hepes-KOH (pH 7.5), 150 mM NaCl, 1% Triton X-100, 10% glycerol, 1 mM EDTA, 1 mM phenylmethylsulfonyl fluoride, 4 mg/ml aprotinin, 2 mg/ml pepstatin, 100 mM NaF and 10 mM tetrasodium pyrophosphate. 10 mg brain extracts were separated in 10% SDS-polyacrylamide gel and transferred to nitrocellulose membrane (Amersham Bioscience) at 100V for 1h. The membrane was blocked with 5% nonfat milk in TBS (Tris-buffered saline) and 0.1% Tween 20 for 1h at room temperature, followed by 2h incubation at room temperature with anti-phospho-CaMKIIα (1:2000 dilution in TBS, 2.5% bovine serum albumin and 0,1% Tween 20, Millipore), anti-Actin or anti-Zif268 (1:500 dilution in TBS, 2.5% bovine serum albumin and 0,1% Tween 20, Santa Cruz Biotechnology). After three washings with 0.1% Tween 20 in TBS, bound antibodies were detected with horseradish peroxidase-conjugated goat anti-rabbit IgG (1:2000 dilution in TBS, 2.5% nonfat milk and 0,1% Tween 20, Santa Cruz Biotechnology, Inc.). After incubation for 1h at room temperature and washings with TBS, the bound antibodies were detected using autoradiographic enhanced ECL chemiluminescence system (Amersham Bioscience). The membranes were neutralized with 15% H_2_O_2_ for 30 min at room temperature and after washings incubated with anti-CaMKIIα which recognize both phosphorylated and non-phosphorylated proteins (1:2000).
