# Supplementary material for: Synaptic Homeostasis and Restructuring across the Sleep-Wake Cycle
Source: PLoS Comput Biol. 2015 May 28;11(5):e1004241. doi: 10.1371/journal.pcbi.1004241 (PMC4447375; doi:10.1371/journal.pcbi.1004241)
Supplement: S4 Text — (DOCX) [file pcbi.1004241.s004.docx]

## Text S4. Quantification of protein levels.

ImageJ software was used to perform densitometric analysis of pCaMKIIα, total CaMKIIα, and Actin staining of dorsal hippocampal cell layers (DG, CA3 and CA1) sampled between -3.0 and -3.5 AP from Bregma; adjacent white matter OD values were subtracted from each measurement. Cells stained for Zif-268 were also counted using a Stereo Investigator system (BMF Bioscience, USA). Labeled cells were counted inside grids of 50X50 μm automatically selected by the software upon the manual delimitation of the cellular layers. Individual values were normalized to average group values and assessed with Kruskal-Wallis (α=0.05) and Dunn’s post-hoc test. Protein levels in the dentate gyrus, CA3 and CA1 fields of the dorsal hippocampus were averaged into a pooled hippocampal measure.
