# Supplementary material for: Synaptic Homeostasis and Restructuring across the Sleep-Wake Cycle
Source: PLoS Comput Biol. 2015 May 28;11(5):e1004241. doi: 10.1371/journal.pcbi.1004241 (PMC4447375; doi:10.1371/journal.pcbi.1004241)
Supplement: S5 Text — (DOCX) [file pcbi.1004241.s005.docx]

## Text S5. Electrophysiological quantifications.

The last 15 min before killing were used for all electrophysiological quantifications. Power in the delta and theta spectral ranges was obtained using a single finite impulse filter with frequency settings of 0.5 to 4.5 Hz and 4.5 to 12 Hz, respectively, both computed over raw hippocampal LFP. Amplitude values were then squared to obtain energy estimate, summed over the 15 minutes period, and finally divided by the sampling frequency. The algorithm for spindle detection and quantification comprised two finite impulse response digital filters in series, capable of detecting spindles from the parietal LFP lead. The routine first separated spindles from background with a 7–14 Hz band-pass filter. Next it extracted spindle oscillation envelopes with durations longer than 0.5 seconds by applying a low-pass filter (cutoff frequency at 2 Hz) to the absolute value of the previous step output. Envelopes with amplitude >5 standard deviations computed for the whole recording were considered spindle events.
