# Supplementary material for: Synaptic Homeostasis and Restructuring across the Sleep-Wake Cycle
Source: PLoS Comput Biol. 2015 May 28;11(5):e1004241. doi: 10.1371/journal.pcbi.1004241 (PMC4447375; doi:10.1371/journal.pcbi.1004241)
Supplement: S6 Text — (DOCX) [file pcbi.1004241.s006.docx]

## Text S6. Canonical hippocampal-cortical network (Model 2).

**Neuron model of the canonical network:** Neurons were implemented as one-compartment leaky integrate and fire units. Instantaneous membrane potential, , of neuron $i$ was related to the feedforward current evoked by the EPSP, , the global feedback current evoked by the IPSP, , and an after hyperpolarization current,

(1)

Where R_N_ = 100 MΩ is the membrane resistance and τ_N_ = 20 ms the membrane time constant. Whenever the membrane potential reached a threshold level, V_TH_= -50 mV, the neuron emitted an action potential, , and the membrane potential was reset to a rest potential, V_REST_= -60 mV:

(2)

$S_{N}^{i}\left[ t \right]=\left\{ \begin{matrix} V_{N}^{i}\left[ t \right]>V_{TH}, & 1 \\ else, & 0 \end{matrix} \right.$ (3)

An after hyper-polarization current ( ) was released following a spike of neuron $i$ with amplitude of A_AHP_ = -2 nA and time constant of τ_AHP_ = 1.7 ms in an exponential decay.

The feed-forward excitatory current, , was computed by the difference between the excitatory potential, V_FF_=0 mV and the membrane potential modulated by the sum of influence of single input events weighted by the specific input-neuron synapse conductance, W_ij_[t]. Single events evoked EPSP was subject to an exponential decay with time constant of τ_FF_ = 5 ms:

(4)

The feedback inhibitory current, I_FB_[t], was obtained as a delayed (d_FB_= 3.3 ms) exponential decay function of amplitude A_FB_ = -20 nA and decay of τ_FB_ = 3 ms and was triggered by the first spike in the network after a period of 10 ms after previous IPSP release, t_last_[t]:

(5)

(6)
