# Supplementary figures and images for: Synaptic Homeostasis and Restructuring across the Sleep-Wake Cycle
Source: PLoS Comput Biol. 2015 May 28;11(5):e1004241. doi: 10.1371/journal.pcbi.1004241 (PMC4447375; doi:10.1371/journal.pcbi.1004241)

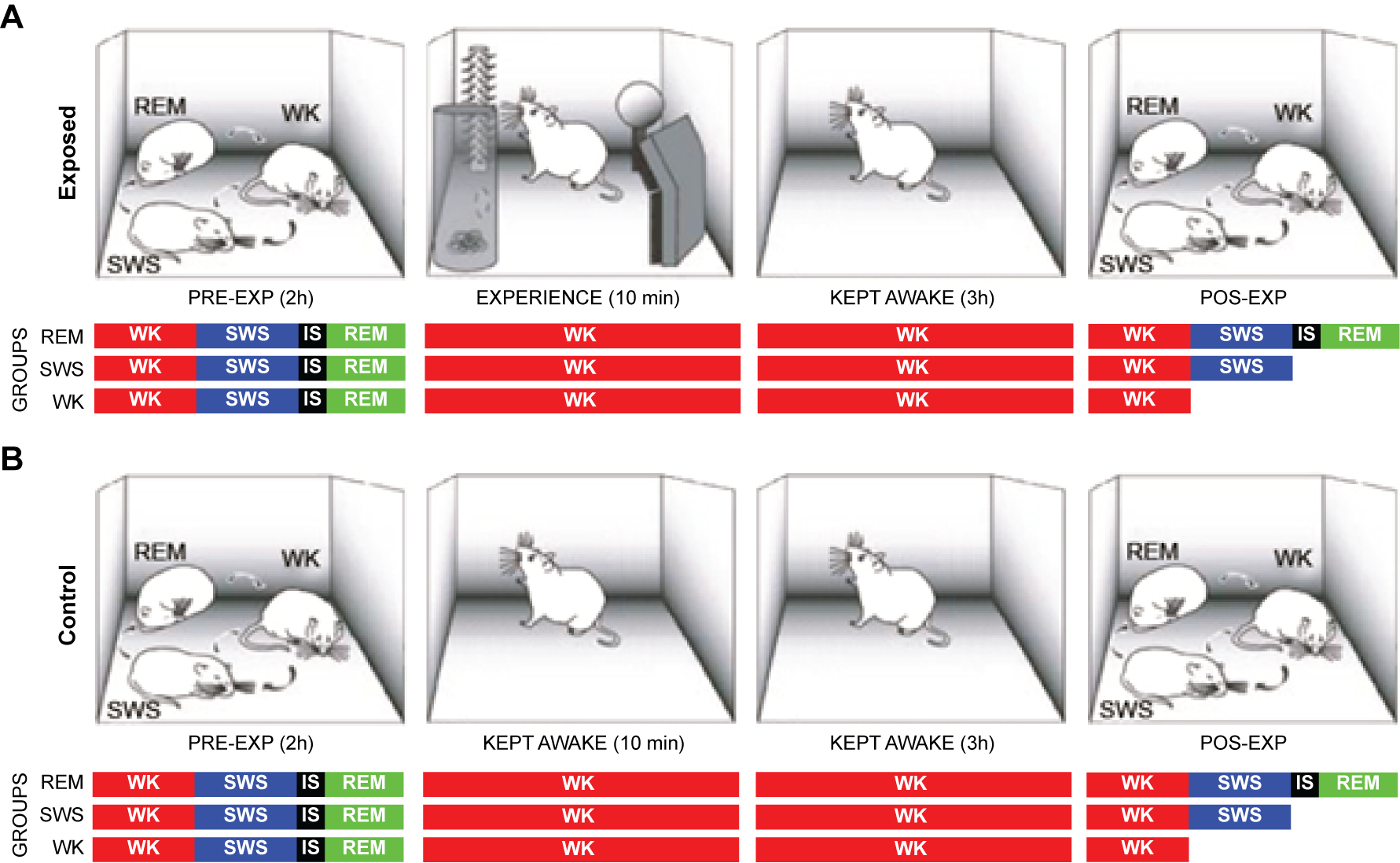

Supplement: S1 Fig — All animals showed intense exploratory activity during exposure to the novel objects (40–90% of the time spent in active exploration, mean 70%). Following novel object exploration (A) or not (B), rats were kept awake for 3 hours and were then allowed to sleep. After one criterion episode of WK, SWS or REM, rats were immediately killed, the brains were frozen and processed for immunohistochemistry. IS represents the intermediate sleep state that separates SWS from REM [43]. Drawings adapted from [20]. (TIF) [file pcbi.1004241.s007.tif]

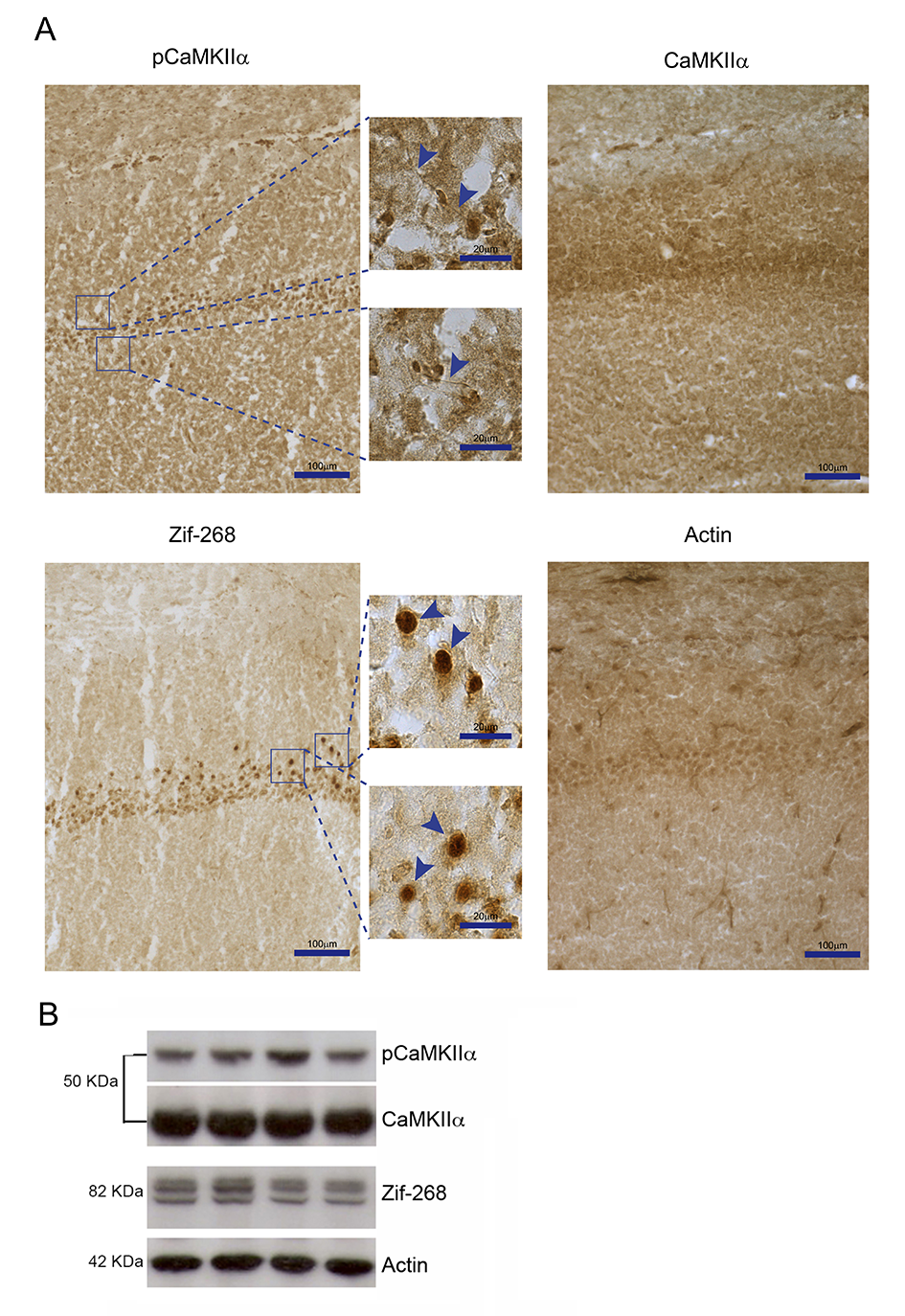

Supplement: S2 Fig — (A) Labeling patterns in the dorsal CA1 field of the hippocampus for pCaMKIIα, total CaMKIIα, Zif-268 and Actin. The anti-pCaMKIIα antibody led to cytoplasmic labeling, marking both the soma and the neuropil (arrows in top center panels). The anti-Zif-268 antibody produced nuclear labeling (arrows in bottom center panels). (B) Antibody specificity was confirmed in immunoblots using hippocampus extracts. (TIF) [file pcbi.1004241.s008.tif]

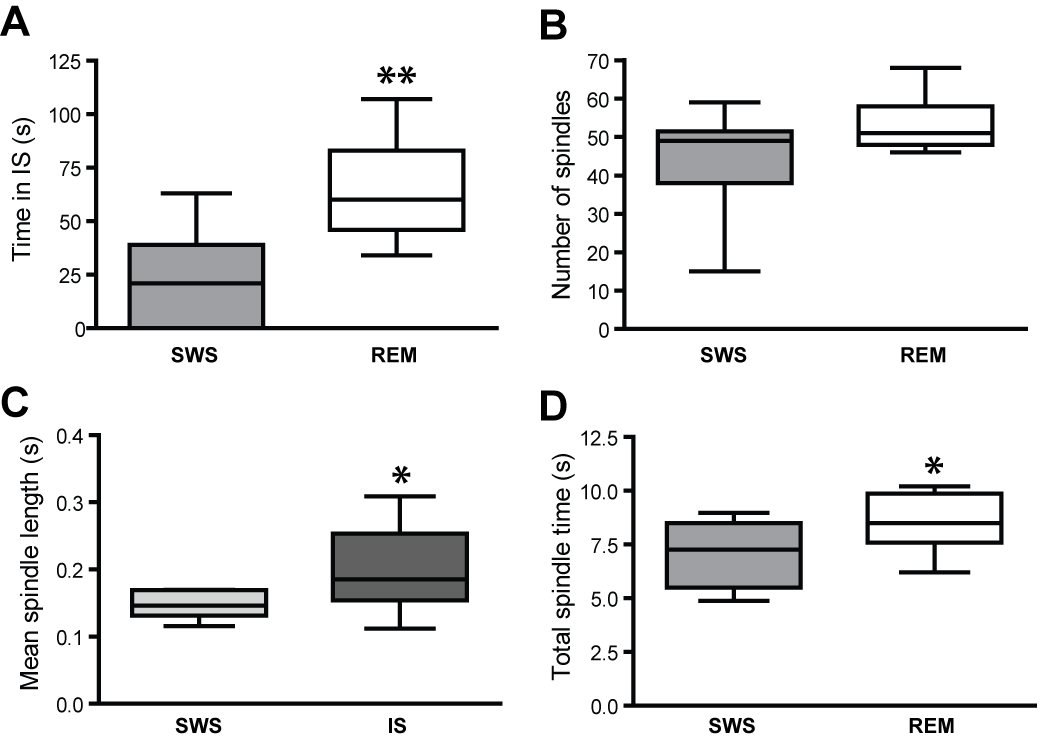

Supplement: S3 Fig — (A) REM animals spent significantly more time in IS than SWS animals. (B) REM animals did not show a significantly greater count of spindles than SWS animals. (C) In the REM group, spindles lasted significantly longer in IS than in SWS. (D) Overall, animals in the REM group spent significantly more time with spindle activity than animals in the SWS group. All results for data collected from the parietal lead (* p < 0.05, *** p < 0.01, unpaired t test). (TIF) [file pcbi.1004241.s009.tif]

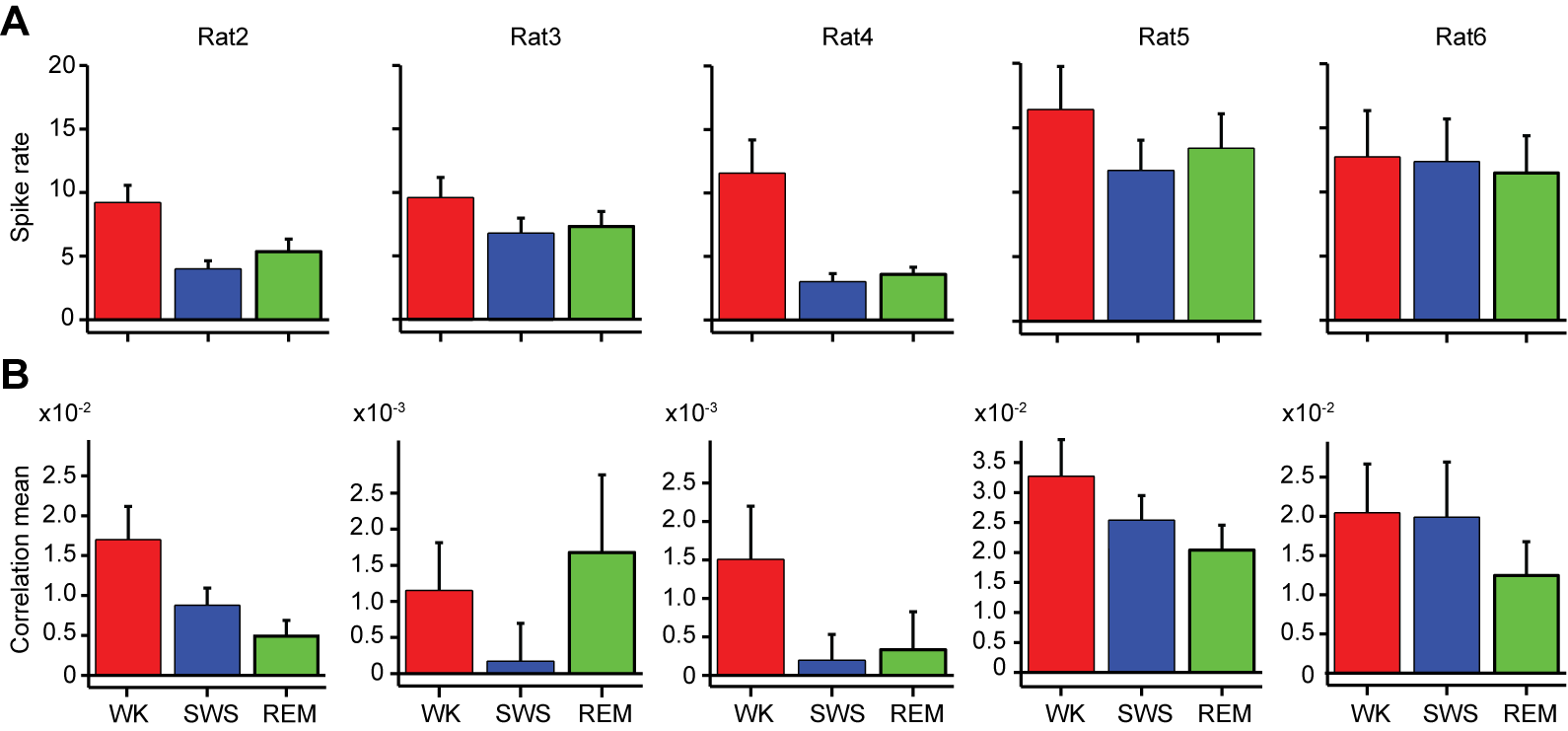

Supplement: S4 Fig — (A) Spike rates and (B) Pearson's linear correlation mean and variance for spiking during WK, SWS or REM. Data recorded from the CA1 field of the hippocampus of 5 rats (columns A2—A6). (TIF) [file pcbi.1004241.s010.tif]

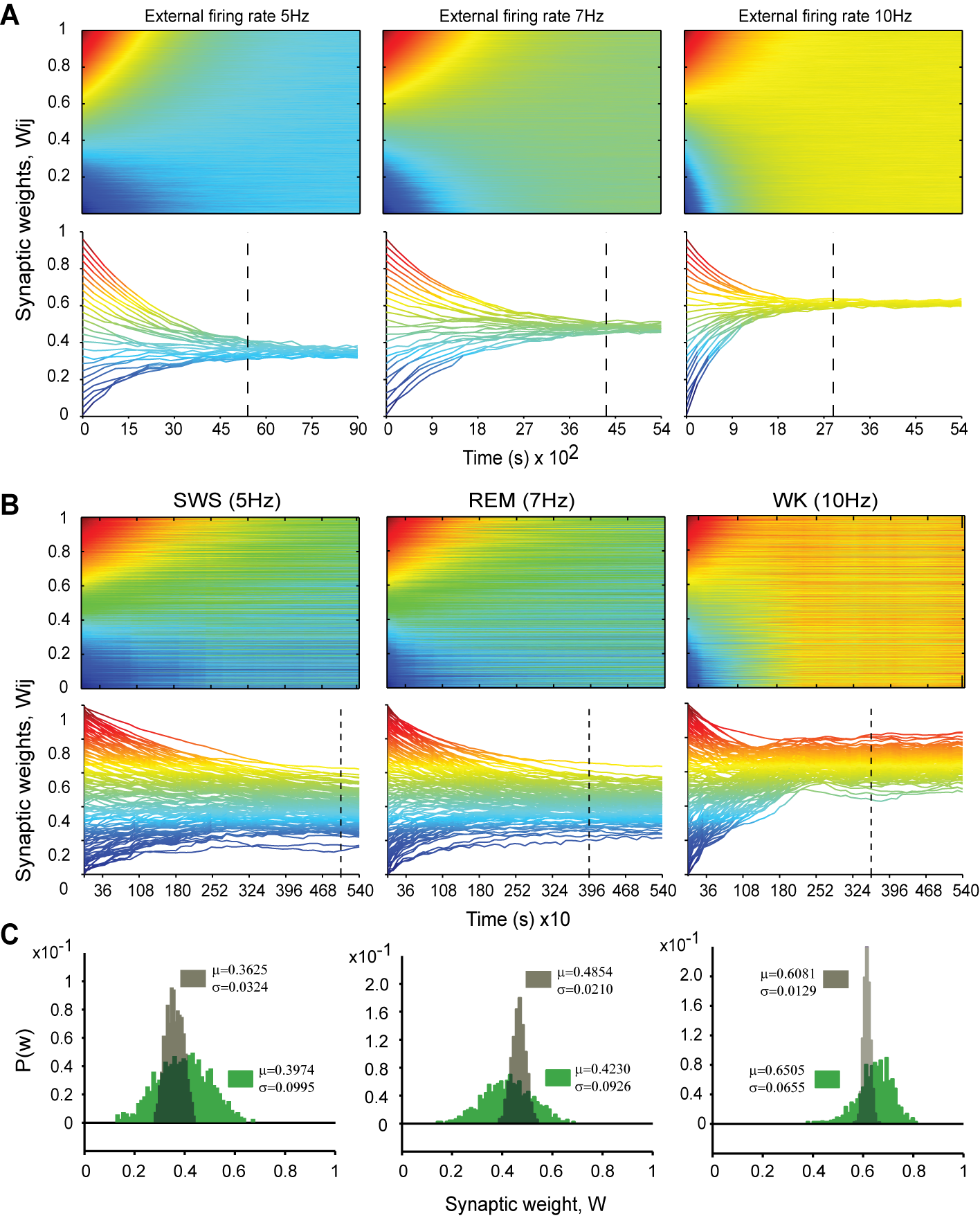

Supplement: S5 Fig — (A) Synaptic weight variation over time (w(t)) when the network is exposed to Poisson inputs at 5Hz, 7Hz, or 10Hz (columns). First row: colored areas represent the dynamics of the synaptic weight values. Second row: synaptic weight trajectories over time allow for a better visualization of the convergence points in the vertical axes. (B) Simulations run for separate sleep states. Real episodes of WK, SWS and REM were concatenated, and their corresponding spike activity was used to feed the network. Dynamics of the synaptic weight values in the first column, synaptic weight trajectories in the second column. In (A) and (B) simulations, the initial synaptic weight values were uniformly distributed in the range [0…1], and the colors attributed for each w ij values change over time, reflecting synaptic weight changes. (C) Final synaptic weight distributions (green area) obtained at the convergence time point (dash black line in panel B) for each stage (WK, SWS and REM, from left to right panels). The corresponding final distributions for Poisson inputs are also displayed (gray area, same mean firing rate as in panel A). (TIF) [file pcbi.1004241.s011.tif]

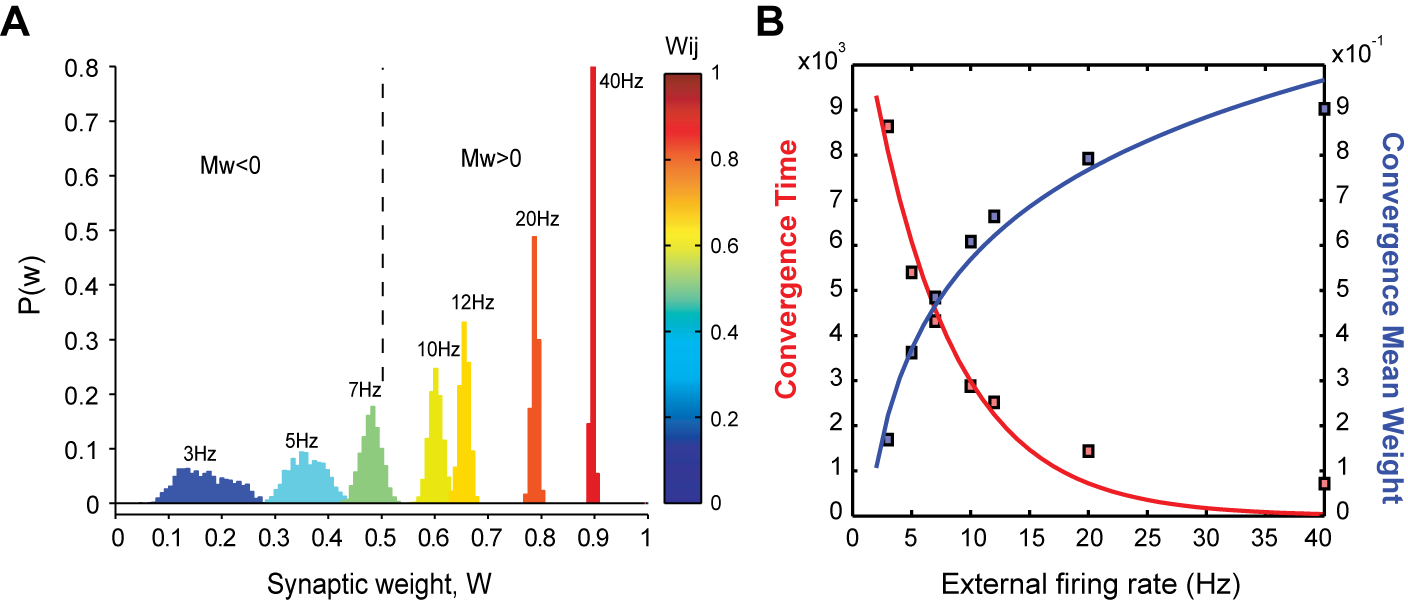

Supplement: S6 Fig — (A) Weight distributions at the convergence time point for every Poisson inputs rate simulated. Firing rates ≤ 7Hz or >7Hz caused net synaptic downscaling (Mw<0) or up-scaling (Mw>0), respectively. (B) As external rates increase, convergence time decreases exponentially (red), and the mean weight achieved at the time of convergence increases logarithmically (blue). (TIF) [file pcbi.1004241.s012.tif]

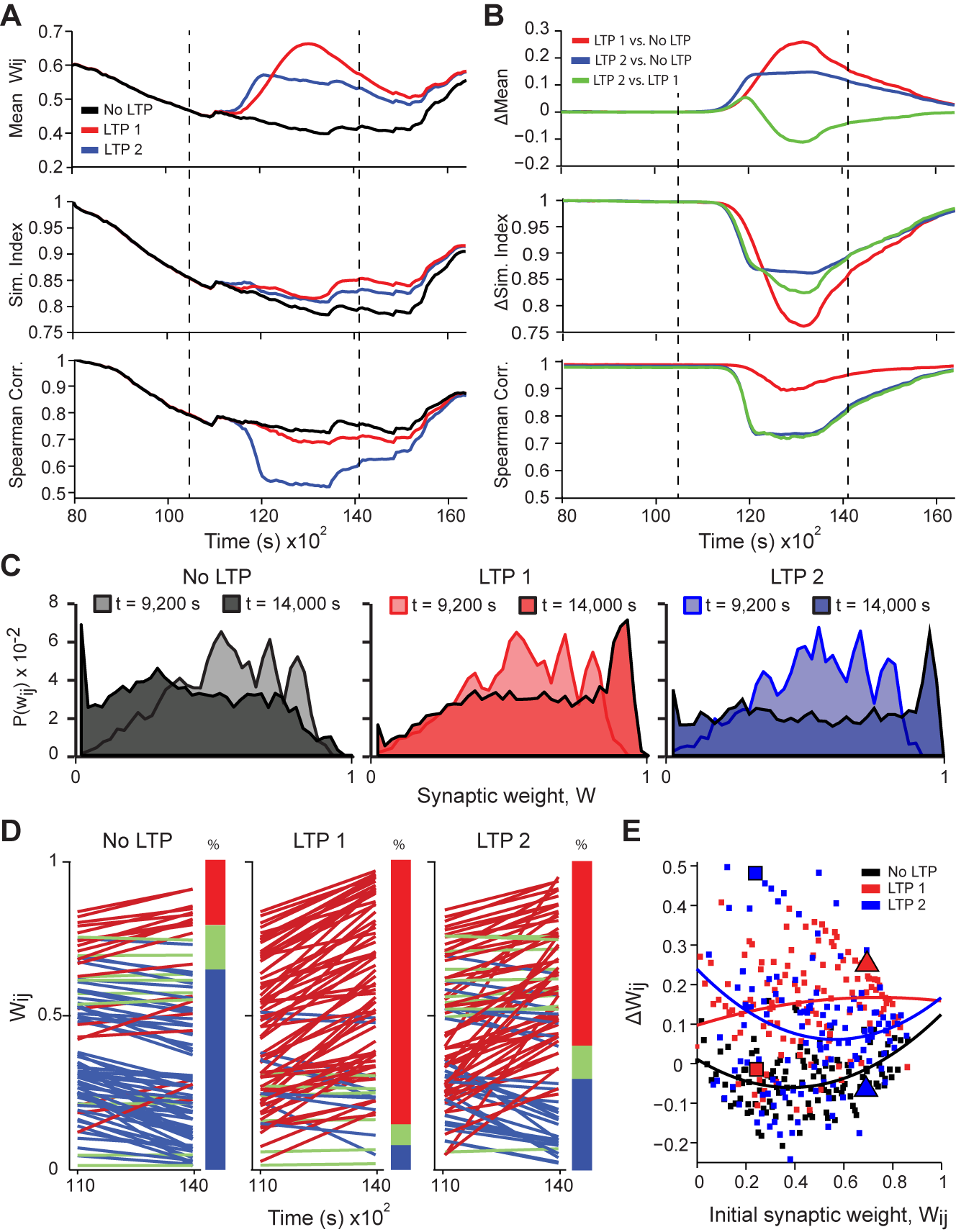

Supplement: S7 Fig — (A) Top LTP1 full SWS model panel shows mean synaptic weights for simulations without LTP (black), with synchronization-based LTP during SWS (red), and with LTP based on synaptic trajectories at the SWS/REM transition (blue). The middle panels show the similarity index over time, comparing the current distribution of wij(t) values at the time t with the initial distribution of w ij(0). The bottom panel shows Spearman´s correlations over time, comparing the distribution of wij(t) with wij(0). (B) Subtractions of mean weights (ΔMean) between LTP and non-LTP models. For A and B panels, dashed lines indicate the time range [10,500s to 14,100s] during which the LTP Gaussian was applied, i.e. boundaries of LTP simulation. (C) Synaptic weight distributions before (9,200s, light colors) and after LTP during sleep (14,000s, dark colors). (D) Changes in synaptic weight values for the initial (11,000s) and last (14,000s) time-points of LTP simulation. Red, green and blue lines represent positive, near-zero and negative slopes, with corresponding percentages indicated by the color bar on the right. (E) Initial synaptic weight values (x axis) versus difference between initial and last time-points of LTP simulation (ΔWij, y axis). Compare the results of the non-LTP model (black) with LTP models 1 and 2 (red and blue, respectively). The curves are quadratic fits. Less of the weak synaptic connections were selected for potentiation in LTP model 2 than in the case of LTP model 1 (10% and 16%, respectively). Notwithstanding, the former were more potentiated overall (S7E Fig, blue curve). There was a net increase in mean synaptic weight value (S7A Fig, top panel, blue curve), and increased spreading towards high synaptic weight values, but with a preservation of low synaptic weights as well (S7C Fig, blue with black edge distribution). A total of 36% of the synapses underwent down-scaling (S7D Fig, right panel, blue bar). Net up-scaling of synaptic weights also occurred for LTP model [file pcbi.1004241.s013.tif]

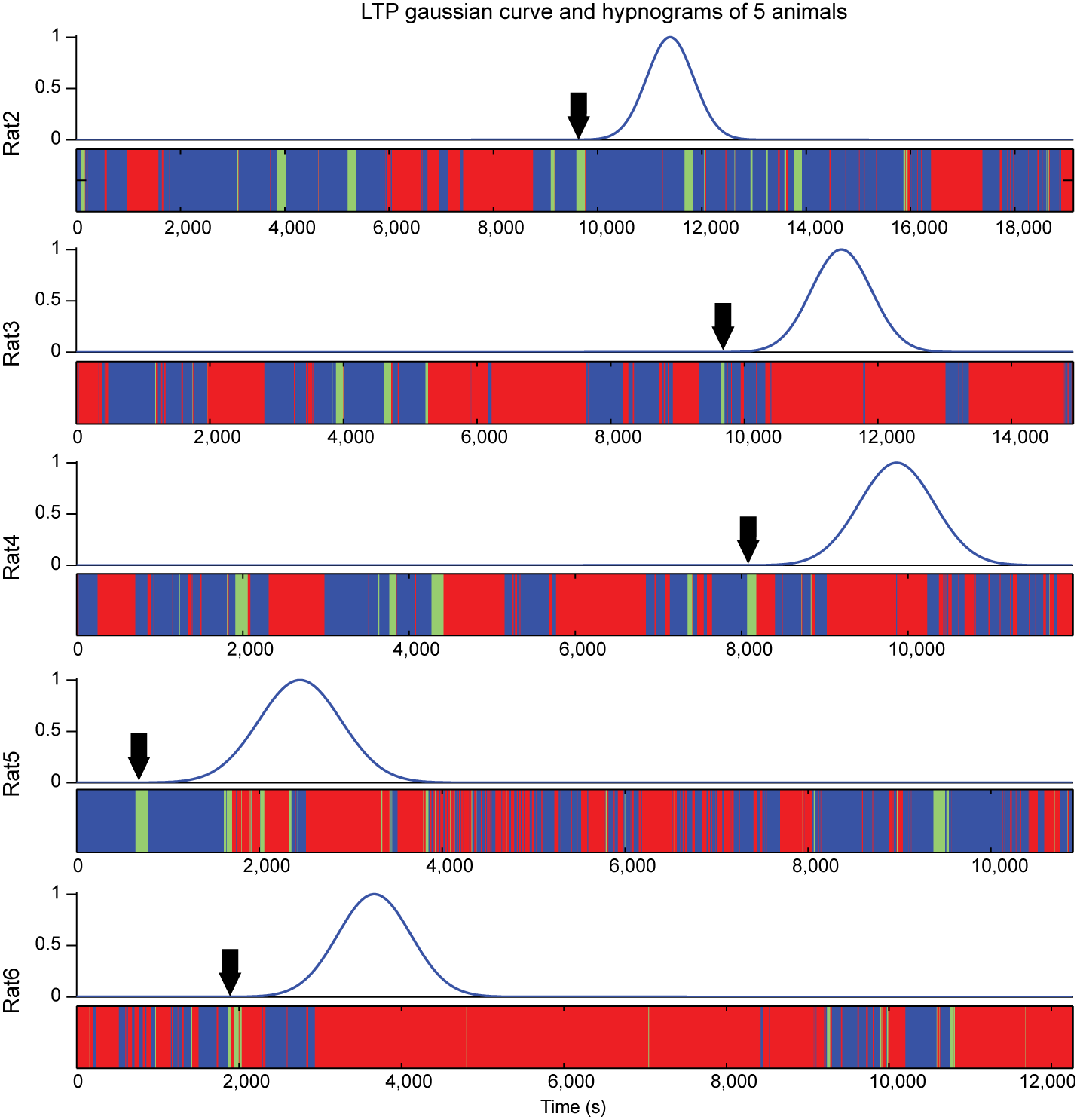

Supplement: S8 Fig — LTP Gaussian curve profiles over real sleep-wake cycles (hypnograms). Black arrows indicates the LTP onsets; n = 5 rats, rows Rat2—Rat6. (TIF) [file pcbi.1004241.s014.tif]

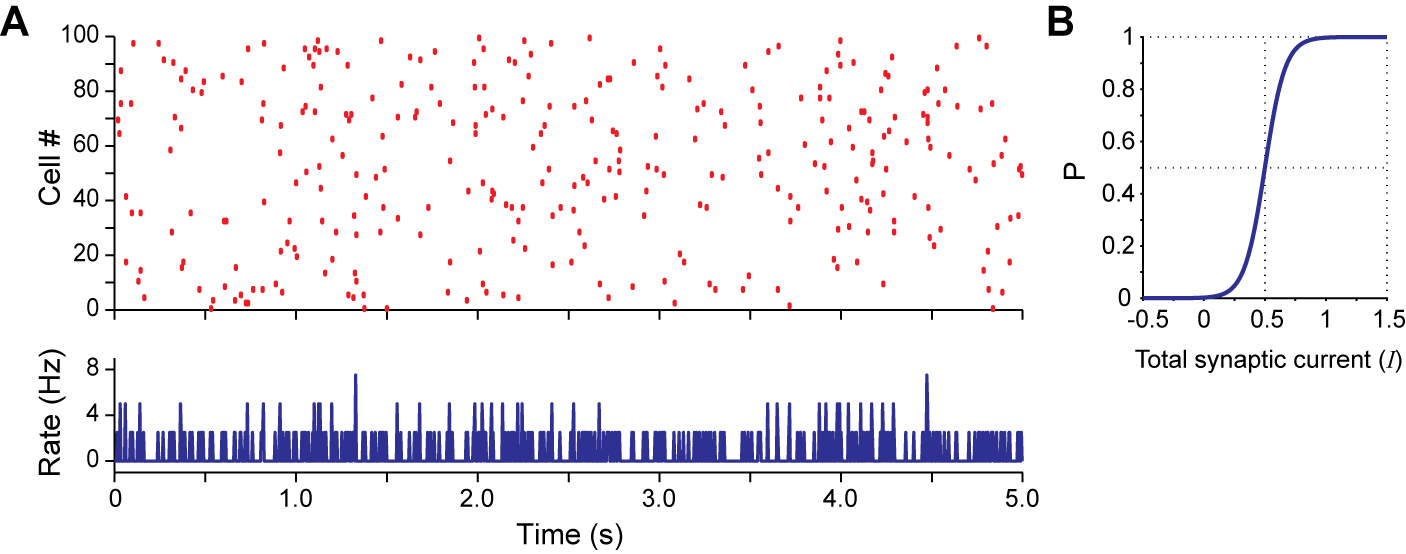

Supplement: S9 Fig — (A) Spike rastergram and its corresponding average population firing rate (network size = 100 neurons). (B) Adjusted sigmoid function used as the probability (P) to update neuron state (see Material and Methods) (TIF) [file pcbi.1004241.s015.tif]

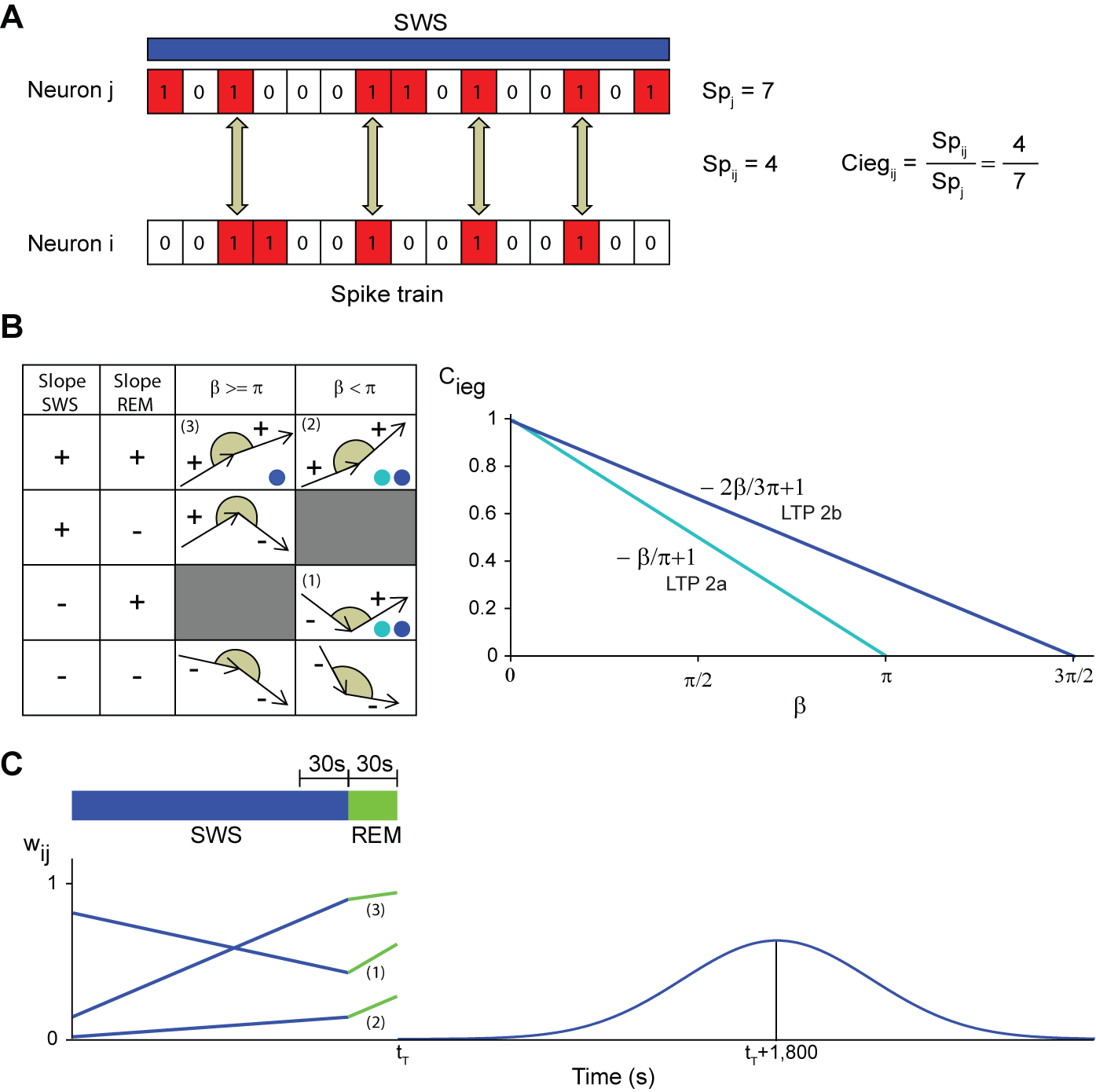

Supplement: S10 Fig — (A) LTP1 was based on spike synchronization during SWS. We counted the spikes when the pre (j) and post (i) synaptic neurons fired simultaneously (represented by Sp ij) over the amount of spikes of the pre-synaptic neuron j (represented by Sp j). This was calculated during a period of SWS previous to the REM stage. (B) In LTP2, Cieg ij varied linearly according to the angle β formed by the W ij (t) values at the SWS➔REM transition. The left panel shows a contingency table of the possible changes in synaptic trajectory slopes at the SWS➔REM transition. The right panel shows two variations of the model, based on different relations between β and Cieg. The most permissive model (dark blue line) applied LTP to all positive slopes during REM, irrespective of the SWS slope (cases 1, 2 and 3). The more restrictive model (light blue) applied LTP only when the REM slope was both positive and larger than the SWS slope (β<π; cases 1 and 2 but not 3). (C) Schematic representation of LTP2, showing the relationship between short-term changes in synaptic trajectory at the SWS➔REM transition and the long-term changes in synaptic weights (LTP), which followed a Gaussian curve triggered at time tT with peak at tT+ 1,800s. (TIF) [file pcbi.1004241.s016.tif]
